# Naturally Occurring Functional Ingredient from Filamentous Thermophilic Cyanobacterium *Leptolyngbya* sp. KC45: Phytochemical Characterizations and Their Multiple Bioactivities

**DOI:** 10.3390/antiox11122437

**Published:** 2022-12-09

**Authors:** Kittiya Phinyo, Khomsan Ruangrit, Jeeraporn Pekkoh, Yingmanee Tragoolpua, Thida Kaewkod, Kritsana Duangjan, Chayakorn Pumas, Nakarin Suwannarach, Jaturong Kumla, Wasu Pathom-aree, Wenhui Gu, Guangce Wang, Sirasit Srinuanpan

**Affiliations:** 1 Department of Biology, Faculty of Science, Chiang Mai University, Chiang Mai 50200, Thailand; 2 Science and Technology Research Institute, Chiang Mai University, Chiang Mai 50200, Thailand; 3 Research Center of Microbial Diversity and Sustainable Utilization, Faculty of Science, Chiang Mai University, Chiang Mai 50200, Thailand; 4 Key Laboratory of Experimental Marine Biology, Center for Ocean Mega-Science, Institute of Oceanology, Chinese Academy of Sciences, Qingdao 266071, China; 5 Laboratory for Marine Biology and Biotechnology, Qingdao National Laboratory for Marine Science and Technology, Qingdao 266000, China

**Keywords:** bioactivity, cyanobacteria, ethanolic extract, functional ingredient, phytochemicals

## Abstract

Cyanobacteria are rich in phytochemicals, which have beneficial impacts on the prevention of many diseases. This study aimed to comprehensively characterize phytochemicals and evaluate multifunctional bioactivities in the ethanolic extract of the cyanobacterium *Leptolyngbya* sp. KC45. Results found that the extract mainly contained chlorophylls, carotenoids, phenolics, and flavonoids. Through LC–ESI–QTOF–MS/MS analysis, 38 phenolic compounds with promising bioactivities were discovered, and a higher diversity of flavonoids was found among the phenolic compounds identified. The extract effectively absorbed the harmful UV rays and showed high antioxidant activity on DPPH, ABTS, and PFRAP. The extract yielded high-efficiency inhibitory effects on enzymes (tyrosinase, collagenase, ACE, and α-glucosidase) related to diseases. Interestingly, the extract showed a strong cytotoxic effect on cancer cells (skin A375, lung A549, and colon Caco-2), but had a much smaller effect on normal cells, indicating a satisfactory level of safety for the extract. More importantly, the combination of the DNA ladder assay and the TUNEL assay proved the appearance of DNA fragmentation in cancer cells after a 48 h treatment with the extract, confirming the apoptosis mechanisms. Our findings suggest that cyanobacterium extract could be potentially used as a functional ingredient for various industrial applications in foods, cosmetics, pharmaceuticals, and nutraceuticals.

## 1. Introduction

Over the course of the last several decades, there has been a gradual but steady rise in people’s understanding of the influence that one’s food may have on one’s overall health. As time has progressed, the idea of food has evolved to include the possibility of avoiding a variety of nutrition-related disorders and enhancing both physical and mental health. The concept of “Let food be thy medicine and medicine be thy food”, first proposed by Hippocrates roughly 2500 years ago, is gaining popularity today as a result of the growing awareness of the importance of eating nutritiously [1]. According to the World Health Organization (WHO), especially in developed nations, dietary patterns and lifestyle behaviors are the primary modifiable risk factors that contribute to the development of certain chronic illnesses such as diabetes, hyperpigmentation, xerosis, and hypertension, among others [2]. Approximately 80% of the world’s population relies on medicines derived from plants in order to treat or prevent illness. It is reasonable to state that up to 50% of all pharmaceuticals now on the market may trace their ancestry back to natural products, even though different estimations will provide different results depending on what exactly is meant by the term “drug derived from a natural product” [3]. Every day, new compounds derived from medical plants that have the potential to be bioactive are discovered; however, only a few of these molecules are investigated to determine whether or not they are suitable for use as pharmaceuticals.

Other natural sources, such as macroalgae, microalgae, and cyanobacteria, are employed as active ingredients in the nutraceutical, cosmetic, and pharmaceutical sectors, in addition to medicinal plants, which are commercially accessible for preventing different ailments. They have a high concentration of bioactive phytochemical substances, particularly pigments and phenolic compounds, both of which are known to be beneficial to human health [1,4,5]. Pigments, such as chlorophylls and carotenoids, are examples of hydrophobic pigments that are commonly present in biomass [6]. Phenolic compounds (polyphenols) are a diverse group of secondary bioactive substances that include the hydroxybenzoic acids (protocatechuic, p-hydroxybenzoic, and syringic), hydroxycinnamic acids (caffeic, p-coumaric, and ferulic), flavan-3-ols (catechin and epicatechin), flavonoids (catechin, epicatechin, quercetin, and apigenin), glycosides, and proanthocyanidins [5]. Because phytochemicals have antioxidant characteristics, and there is evidence that they participate in numerous biological processes, such as those that are cardioprotective, anti-inflammatory, anticarcinogenic, antibacterial, and antiviral [1], there is an increased interest in the study of phytochemicals.

Cyanobacteria (also known as “blue-green algae”) are a great source of bioactive phytochemical substances that are good for our health because they have a lot of pigments and polyphenols [6]. Cyanobacteria are a large and varied group of photosynthetic prokaryotes, with an estimated 8000 species scattered over 150 genera. These organisms occupy a broad range of habitats, from aquatic to terrestrial, and from temperate to tropical to polar locations [7]. Many of the secondary metabolites produced by cyanobacteria are powerful antioxidants that may neutralize reactive oxygen species (ROS). These include polyphenols such as phenolic acids and flavonoids, as well as pigments such as chlorophylls and carotenoids [8]. Due to the higher yields and the use of non-arable land in cultivation, cyanobacteria are said to be superior and an excellent source of health-promoting bioactive compounds compared with other edible plants [4]. There has been a lot of research that has looked at the phytochemical production of different cyanobacteria species, including *Nostoc* spp. [6], *Aphanizomenon* spp. [8], *Phormidium* spp. [5], and *Spirulina* spp. [9]. The phytochemical profiling of cyanobacteria is rather similar to that of medicinal plants and other types of algae; in particular, it is predominately made up of pigments and polyphenols [8]. In fact, a wide variety of bioactivities have been described for these compounds. Some examples are antihyperlipidemic [10], antiobesity [11], antidiabetic [12], antioxidant [5,6], anti-inflammatory [13], antiviral [14], antibacterial [15], antitumoral [13], antiallergic [16], and neuroprotective [17]. Hence, cyanobacteria have gained interest as a possible source of phytochemicals with wide-ranging practical uses. Nevertheless, cyanobacteria, specifically *Leptolyngbya* sp., have not been studied in great depth in terms of the sort or kind of polyphenols that are present or their qualities. This is something that might add value to their exploitation, and perhaps allow for their better usage.

Cyanobacteria can have different biofunctional potentials depending on factors such as the species, the season, the temperature, the environment, the salinity of the water, the quantity of sunshine, the harvesting time, and even the collecting region [5]. Additionally, variations in the concentration of a solvent as well as the polarity of the solvent play significant roles in the process of extracting phytochemical substances [1]. On the cyanobacterium, *Leptolyngbya* sp., that was chosen for this investigation, there is a paucity of information regarding an appropriate solvent and solvent and its concentration for the purpose of extracting bioactivities [18,19]. Furthermore, since climate (global warming), water salinity, pollution, and other factors are always shifting, we consider it necessary to periodically re-evaluate phytochemicals and their functions. There is a lack of data on the proportion of phytochemicals in *Leptolyngbya* sp. cultured in Thailand. In addition, the phytochemicals found in Thai *Leptolyngbya* extract have not been analyzed, and it is unknown whether they possess any of the critical functional (antioxidant, enzyme inhibitory activity, and cytotoxicity) or spectrum features. Due to a lack of scientific knowledge and promotion of the cyanobacteria’s untapped potential, they have not caught on with the Thai public or been used to their fullest extent. Hence, they are rarely included in commercial biofunctional products with added value.

In the literature, Mahanil et al. [20] were successful in cultivating *Leptolyngbya* sp. (strain KC45) in an open pond at temperatures ranging from 30 to 45 °C and high alkaline pH levels in the range of 8–10. Because of this, they were able to create an adequate amount of biomass (raw material) for sale, in addition to producing thermostable pigments for use in industrial applications. They also discovered that the toxic substance known as microcystin was not present in the *Leptolyngbya* sp. KC45 extract, indicating that it possesses features that are suitable for future applications. However, the characterization of phytochemicals in strain KC45 has not yet been studied, despite the fact that *Leptotynbra* spp. possesses a wide variety of phytochemicals that are useful. We, therefore, used the cyanobacterium *Leptolyngbya* sp. KC45. in order to determine whether or not there is a potentially viable use.

Hence, the objective of this work was to investigate the possibility of employing the ethanolic extract from the cyanobacterium *Leptolyngbya* sp. KC45 as a functional ingredient. For this purpose, the extract’s phytochemical profiles were first evaluated by measuring its ultraviolet–visible (UV–Vis) absorption potential and determining the amounts of carotenoids, chlorophylls, polyphenols, and flavonoids present. The polyphenols in the ethanolic extract that were responsible for the functional characteristics were identified through LC–MS/MS analysis. In the second step, the extract was tested for its antioxidant characteristics and its ability to inhibit several enzymes to prove its in vitro health benefits; these enzymes included tyrosinase, collagenase, angiotensin-converting enzyme (ACE), and α-glucosidase. As a result of an increase in the number of patients suffering from skin disorders, hypertension, xerosis, and diabetes, these tests have taken on an increased level of significance in today’s medical landscape. Lastly, the in vitro cytotoxicity of the extract against skin cancer A375 cells, lung cancer A549 cells, and colon cancer Caco-2 cells were carefully determined and reported for the first time. Accordingly, we postulated that *Leptolyngbya* extract would be useful as a functional ingredient and in the treatment of a wide range of chronic diseases.

## 2. Materials and Methods

### 2.1. Cyanobacterial Biomass

*Leptolyngbya* sp. KC45 isolated from a thermal spring at a temperature of 45 °C [21] was obtained from the Algal and Cyanobacterial Research Laboratory, Chiang Mai University, Thailand. The cyanobacteria were cultured in 2000 L of D-medium in an open raceway pond under ambient conditions (12 h photoperiod, 25.9–62.9 µmol m^−2^ s^−1^ photosynthetically active radiation, 24.1–27.6 °C temperature range) for 30 days. After the end of cultivation, the cyanobacteria were cleaned with tap water and shade dried at 60 °C using a hot air oven. The dried cyanobacteria were ground into powder using a high-speed multifunction mill grinder (ARTC, China) and then stored at −20 °C before use.

### 2.2. Extract Preparation

The cyanobacterial extracts were extracted using microwave-assisted ethanol extraction according to the method described by Rodriguez-Jasso et al. [22] with minor modifications. Briefly, dried biomass was suspended in the ethanol and placed into the extraction vessel at an alga/water ratio of 1:10 (*w*/*v*). The suspension was subjected to microwave irradiation (Sharp R-221F-K, Thailand) under the operating conditions at a power of 800 W and frequency of 2450 MHz for 1 min. After irradiation, the vessels were immediately cooled in an ice bath and the suspensions were filtrated through Whatman filter paper No. 1 to obtain the supernatant. After that, the supernatant was heated in a rotary evaporator at 40 °C and under decreased pressure so that it could be evaporated to dryness. Then, the extract was lyophilized and stored at −20 °C before use.

### 2.3. Phytochemical Characterization

#### 2.3.1. Determination of Chlorophylls and Carotenoids

The extract was mixed with ethanol at the concentration of 1 mg/mL. The ethanol was used as a blank. Values of spectrophotometric absorbance at 480, 632, 649, 665, 696, and 750 nm wavelength of the mixture were recorded [23]. The chlorophyll a (C_a_), chlorophyll b (C_b_), chlorophyll c (C_c_), chlorophyll d (C_d_), total chlorophyll (C_t_), and carotenoid (C_n_) contents (µg/mL) were calculated using the following equation:

C_a_ = [0.0604 × (A632 − A750)] − [4.5224 × (A649 − A750)] + [13.2969 × (A665 − A750)] − [1.7453 × (A696 − A750)]

   C_b_ = [− 4.1982 × (A632 − A750)] + [25.7205 × (A649 − A750)] − [7.4096 × (A665 − A750)] − [2.7418 × (A696 − A750)]

C_c_ = [28.4593 × (A632 − A750)] − [9.9944 × (A649 − A750)] − [1.9344 × (A665 − A750)] − [1.8093 × (A696 − A750)]

   C_d_ = [− 0.2007 × (A632 − A750)] + [0.0848 × (A649 − A750)] − [0.1909 × (A665 − A750)] + [12.1302 × (A696 − A750)]

C_t_ = C_a_ + C_b_ + C_c_ + C_d_

C_n_ = 4 × (A480 − A750)

The C_a_, C_b_, C_c_, C_d_, C_t_, and C_n_ contents were expressed as mg/g extract.

#### 2.3.2. Determination of Total Phenolics

The total phenolic content (TPC) was spectrophotometrically determined according to the Folin–Ciocalteu reagent method [5]. Briefly, 20 µL of the extract solution was combined with 20 µL of deionized water and 100 µL of Folin–Ciocalteu reagent that had a weight-to-volume ratio of 10%. The mixture was allowed to incubate for 5 min at room temperature (30 ± 1 °C) before being combined with 80 µL of 5% Na_2_CO_3_ solution. At room temperature and in dark conditions, the combination was allowed to incubate for 1 h. A spectrophotometric analysis of the mixture was performed at a wavelength of 765 nm, with gallic acid serving as the reference standard. TPC was reported as mg gallic acid equivalent (GAE) per g of extract.

#### 2.3.3. Determination of Total Flavonoids

The total flavonoid content (TFC) was determined by the aluminum chloride method with some modifications [24]. In a 96-well plate, 80 µL of the extract was combined with 80 µL of 2% aluminum chloride solution that had been diluted with ethanol, and 120 µL of solution that included sodium acetate at a concentration of 50 g/L. The plate was then incubated at room temperature for 2.5 h. After that, the absorbance of the mixture was measured at 440 nm, and quercetin was employed as the standard for comparison. TFC was calculated as mg of quercetin (QE) equivalent per g of extract.

#### 2.3.4. Determination of Ultraviolet (UV) Absorption

The extract was mixed with ethanol at the concentration of 0.33 mg/mL. The ethanol was used as a blank. The mixture was observed for absorbance at 250–700 nm wavelength (particularly, the critical wavelengths of 250 and 280 nm for UV-C range, 290 and 310 nm for UV-B range, 330 and 350 nm for UV-A range were chosen for comparison) using a UV spectrophotometer [6].

#### 2.3.5. Liquid Chromatography–Electrospray Ionization–Quadrupole Time-of-Flight–Mass Spectrometry (LC–ESI–QTOF–MS/MS) Analysis

The phenolic composition of the extract was identified using LC–ESI–QTOF–MS/MS analysis reported by Lomakool et al. [5]. LC–ESI–QTOF–MS/MS analysis was carried out by utilizing an Agilent 1200 series HPLC (Agilent Technologies, Santa Clara, CA, USA) in conjunction with an Agilent 6545 Accurate-Mass Q-TOF LC–MS (Agilent Technologies, Santa Clara, CA, USA) that was connected to an electrospray ionization source (ESI). A Poroshell 120 EC-C18, LC Column (2.1 × 100 mm, 2.7 µm) (Agilent Technologies, Santa Clara, CA, USA) was utilized in order to accomplish the separation. The components of the mobile phase were as follows: water and acetic acid at a ratio of 98:2 (*v*/*v*); eluent A; and acetonitrile, acetic acid, and water at a ratio of 50:0.5:49.5 (*v*/*v*/*v*); eluent B. The following is how the gradient profile was described: 10–25% B (from 0 to 25 min), 25–35% B (from 25 to 35 min), 35–40% B (from 35 to 45 min), 40–55% B (from 45 to 75 min), 55–80% B (from 75 to 79 min), 80–90% B (from 79 to 82 min), 90–100% B (from 82 to 84 min), 100–10% B (from 84 to 87 min), and an isocratic 10% B at the end (from 87 to 90 min). The extract sample was injected at a volume of 6 µL, and the flow rate was set at 0.4 mL/min. The nebulization of nitrogen gas was set at 45 psi with a flow rate of 5 L/min at a temperature of 300 °C, and the flow rate of the sheath gas was set at 11 L/min at a temperature of 250 °C. The voltage of the capillary was set at 3.5 kV, while the voltage of the nozzle was set at 500 V. MS/MS studies were carried out in automated mode with collision energies of 10, 15, and 30 eV for fragmentation. A full mass scan was performed, ranging from *m*/*z* 50 to 1300. MassHunter Workstation software (Metlin_Metabolites_AM_PCDL.cdb) (Agilent Technologies, Santa Clara, CA, USA) was utilized for instrument management, data collecting, and processing, with positive and negative modes of operation utilized for peak detection.

### 2.4. Bioactivity Evaluations

#### 2.4.1. 2,2-Diphenyl-1-Picrylhydrazyl (DPPH) Antioxidant Assay

The DPPH radical scavenging activity was measured in accordance with the methodology outlined by Cheirsilp et al. [25], although with some slight adjustments. In a 96-well plate, 50 μL of DPPH dissolved in methanol at 1.3 mM concentration was combined with 100 μL of the extract solution. The mixture was kept in an incubator at room temperature in the dark for a period of 30 min. The absorbance of the mixture was determined to be 517 nm. Gallic acid was utilized as the standard of reference, and the following equation was applied in order to calculate the scavenging activity (%).

DPPH radical scavenging activity = [(A_control_ − A_sample_)/A_control_] × 100

where the absorbance of the sample, denoted by “A_sample_”, was determined; the absorbance of the control reaction, denoted by “A_control_”, was also determined, using ethanol in place of the sample. The half maximal inhibitory concentration (IC_50_) was calculated as the concentration of the tested sample that inhibited 50% of DPPH radical scavenging. The concentration of the tested sample providing 50% inhibition is estimated by plotting the percent of inhibition against different concentrations of the tested sample. To calculate IC_50_, the various concentrations of the tested sample and the percentage of inhibition were plotted on the *x*- and *y*-axes, respectively, and the data were then expressed mathematically as inhibition curve. The concentration of tested sample that gives 50% inhibitory activity was recorded. The gallic acid equivalent (GAE) per g of extract is the unit of measurement used to report the DPPH activity. 

#### 2.4.2. 2,2′-Azino-Bis (3-Ethylbenzthiazoline-6-Sulfonic Acid) (ABTS) Radical Scavenging Assay

The ABTS radical scavenging activity was measured in accordance with a modified version of the technique described in Ruangrit et al. [26]. The ABTS solution was made up by combining 7 mM of ABTS solution with 2.45 mM potassium persulfate in the appropriate amounts (final concentration). The mixture was kept in the incubator for 12–16 h at room temperature and under dark conditions. After that, the absorbance of the ABTS solution at 734 nm was brought to the desired value of 0.70 ± 0.02 by applying deionized water. Within a 96-well plate, 5 μL of the extract solution and 195 μL of the ABTS solution were combined and stirred together. After incubating the mixture for 10 min at 37 °C, the amount of ABTS that was reduced was measured at 734 nm. Gallic acid was utilized as the standard of reference, and the following equation was applied in order to calculate the scavenging activity (%).

ABTS radical scavenging activity = [(A_control_ − A_sample_)/A_control_] × 100

where the absorbance of the sample, denoted by “A_sample_”, was determined; the absorbance of the control reaction, denoted by “A_control_”, was also determined, using ethanol in place of the sample. The half maximal inhibitory concentration (IC_50_) was calculated as the concentration of the tested sample that inhibited 50% of ABTS radical scavenging. The IC_50_ value was determined in the same manner that was discussed in Section 2.4.1, which can be found above. The gallic acid equivalent (GAE) per g of extract is the unit of measurement used to report the ABTS activity. 

#### 2.4.3. Potassium-Ferricyanide-Reducing Antioxidant Power (PFRAP) Assay

The PFRAP test was carried out in accordance with the procedures described in Lomakool et al. [5], but with minor adjustments. A mixture that consisted of 130 μL of the extract solution, 290 μL of 0.2 M sodium phosphate buffer with a pH of 6.6, and 290 μL of 1% potassium ferricyanide was created by mixing all of these ingredients together. The mixture was kept warm in an incubator for 20 min at a temperature of 50 °C. Following the addition of 290 μL of trichloroacetic acid with a weight-to-volume ratio of 10%, the mixture was centrifuged at 3000 rpm for 10 min. After that, 1 mL of the supernatant solution was combined with 1 mL of distilled water and 200 µL of 0.1% ferric chloride. The absorbance was evaluated at a wavelength of 700 nm, and gallic acid served as the benchmark for comparison. Gallic acid equivalent (GAE) per g of extract is the unit of measurement used to report the PFRAP activity.

#### 2.4.4. Determination of Tyrosinase Inhibitory Activity

Tyrosinase inhibitory activity of the extract was evaluated using L-DOPA as the substrate according to the method described by Pekkoh et al. [27] with minor modifications. To summarize, 40 μL of the extract solution was combined with 80 μL of phosphate buffer (100 mM, pH 6.8) and 40 μL of the tyrosinase enzyme (100 U/mL) in a 96-well microplate. The mixture was then placed in an incubator at 37 °C for 5 min. After that, 40 μL of L-DOPA (1 mg/mL) was added to the mixture, which was then left to incubate at 37 °C for 20 min. At a wavelength of 475 nm, the absorbance of the mixture was measured. Following is the equation that was used to determine the tyrosinase inhibitory activity (%):

Tyrosinase inhibitory activity = [(A − (B − C))/A] × 100

where the absorbance of the control blank, absorbance of the sample, and absorbance of the sample blank are denoted by the letters A, B, and C, respectively. The half maximal inhibitory concentration (IC_50_) was calculated as the concentration of the tested sample that inhibited 50% of tyrosinase enzyme activity. The IC_50_ value was determined in the same manner as discussed in Section 2.4.1, which can be found above. Kojic acid was used as a standard reference, and the tyrosinase inhibitory activity was calculated as mg-kojic acid equivalent (KE) per g-extract.

#### 2.4.5. Determination of Collagenase Inhibitory Activity

Collagenase inhibitory activity of the extract was evaluated using the modified method of Thring et al. [28]. Briefly, following the mixing of the sample (15 µL) with 62.5 µL of Tricine buffer (50 mM, pH 7.5) and 12.5 µL of the collagenase enzyme (0.8 U/mL) in a 96-well microplate, the mixture was left to incubate for 15 min at room temperature. The mixture was then combined with 60 µL of *N*-[3-(2-furyl) acryloyl]-Leu-Gly-Pro-Ala (FALGPA) substrate, and it was allowed to incubate at room temperature for 20 min. The absorbance was measured at a wavelength of 475 nm. The collagenase inhibitory activity, expressed as a percentage, could be determined using the equation below:

Collagenase inhibitory activity = [(A − (B − C))/A] × 100

where the absorbance of the control blank, absorbance of the sample, and absorbance of the sample blank are denoted by the letters A, B, and C respectively. The IC_50_ was calculated as the concentration of the tested sample that inhibited 50% of collagenase enzyme activity. The IC_50_ value was determined in the same manner that was discussed in Section 2.4.1, which can be found above. Epigallocatechin gallate (EGCG) was used as a standard reference, and the collagenase inhibitory activity was calculated as mg-EGCG equivalent (EGCGE) per g-extract.

#### 2.4.6. Determination of Angiotensin-Converting Enzyme (ACE) Inhibitory Activity

Angiotensin-converting enzyme (ACE) inhibitory activity was determined according to the method described by Pekkoh et al. [29]. Briefly, in each well of a 96-well plate, 5 µL of ACE solution with a concentration of 200 mU/mL was added to 31 µL of sodium borate buffer, with a pH of 8.3, that included 0.3 M sodium chloride (SBBS). Subsequently, 10 µL of the sample or SBBS (the control, C) was added. Following the addition of 13 µL of substrate HHL solution with a concentration of 5 mM to the reaction mixture (with a total volume of 59 µL), the reaction was initiated. Two blanks were prepared: one without ACE and inhibitor sample (Bi), and another without ACE and HHL (Bs). Following a 1 h incubation period at 37 °C, 100 µL of 200 mM sodium tetraborate was added to each well, followed by 50 µL of 10 mM sodium sulfite and 50 µL of 3.4 mM TNBS. After that, the mixture continued to be incubated at 37 °C for another 20 min. The absorbance was measured at a wavelength of 420 nm, and the following equation was applied in order to calculate the percentage of ACE inhibitory activity:

ACE inhibitory activity = [(C − Bi) − (S − Bs)/(C − Bi)] × 100

The half maximal inhibitory concentration (IC_50_) was calculated as the concentration of the tested sample that inhibited 50% of ACE activity. The IC_50_ value was determined in the same manner that was discussed in Section 2.4.1, which can be found above. Enalapril was used as a standard reference, and the ACE inhibitory activity was calculated as mg-enalapril equivalent (EE) per g-extract.

#### 2.4.7. Determination of α-Glucosidase Inhibitory Activity

The method of Tanruean et al. [30] was used for measuring α-glucosidase inhibitory activity. Briefly, the sample was incubated with the α-glucosidase solution (30 µL) at 37 °C for 15 min. After adding 70 µL of d-maltose with a concentration of 37 mM, the mixture was heated to 37 °C for 15 min. The reaction was halted by placing the mixture into boiling water for 10 min. The reaction mixture was then mixed with 1 mL of PGO reagent (containing one capsule of PGO enzymes, 100 mL of water, and 1.6 mL of *O*-dianisidine (2.5 mg/mL) solution) and incubated at 37 °C for 15 min. The absorbance was measured at 450 nm. The α-glucosidase inhibitory activity (%) is calculated based on the following equation:

α-glucosidase inhibitory activity = [(A − B)/A] × 100

where A is the absorbance of the control blank, and B is the absorbance of the sample. The IC_50_ was calculated as the concentration of the tested sample that inhibited 50% of α-glucosidase activity. The IC_50_ value was determined in the same manner that was discussed in Section 2.4.1, which can be found above. Acarbose was used as a standard reference, and the α-glucosidase inhibitory activity was calculated as mg-acarbose equivalent (AE) per g-extract.

### 2.5. Cytotoxicity Test

#### 2.5.1. Cytotoxicity of Cancer Cells and Normal Cells

The cytotoxicity of the extract was tested using the MTT assay [26]. Briefly, the cancer cell line (A375 human melanoma cells, A549 human lung adenocarcinoma cells, and Caco-2 human colorectal carcinoma cells) and normal cell line (Vero cells) were precultured under the following conditions: a temperature of 37 °C in 5% CO_2_ for 24 h. Dulbecco’s Modified Eagle Medium (DMEM) supplemented with 10% heat-inactivated fetal bovine serum (FBS), penicillin (100 Units/mL), and streptomycin (100 µg/mL) was used as the culture medium for the precultivation of A549 cells, Caco-2 cells, and Vero cells, while A375 cells were precultured in DMEM plus pyruvate supplemented with 1% HEPES, 10% FBS, penicillin (100 Units/mL), and streptomycin (100 µg/mL). After precultivation, the cells (10^5^ cell/mL) were transferred to 96-well plates and further incubated at 37 °C in 5% CO_2_ for 24 h. Each concentration of extract was prepared and added into each well and then incubated at 37 °C in 5% CO_2_ for 48 h. After that, the treated cells were reacted with 30 μL of solution containing 2 mg/mL of 3-(4,5-dimethylthiazol-2-yl)-2,5-diphenyltetrazolium bromide (MTT), and they were incubated continuously for a period of 4 h. Following incubation, DMSO to the volume of 200 μL was added to each well, and the blue formazan contents were thoroughly mixed. The absorbance levels were measured at 540 nm and 630 nm. The percentage of cell viability was calculated by comparing the relevant values to the cell control based on the following equation:

Cell viability (%) = (A_treated cells_/A_control_) × 100

where the absorbance of culture cells is denoted by the variable A_control_, whereas the absorbance of treated culture cells is denoted by the variable A_treated cells_. The IC_50_ was calculated as the concentration of the tested sample that inhibited 50% of culture cells.

#### 2.5.2. Deoxyribonucleic Acid (DNA) Fragmentation Analysis using DNA Ladder Assay

The DNA ladder assay was used to detect the DNA fragmentation of the cells after treatment with the extract [31]. Briefly, the cancer cells at a concentration of 2 × 10^5^ cells/mL were cultured in 24-well plates, and then incubated at 37 °C in 5% CO_2_ for 24 h. The cells were treated with the extract and further incubated at 37 °C in 5% CO_2_ for 48 h. After having been rinsed three times with phosphate buffered saline (PBS, pH 7.4) and trypsinized with a solution containing 0.05% trypsin-EDTA, the pellet cells were harvested and lysed by adding 30 µL of lysis solution (containing 10 mM Tris-HCl, 2.5 mM EDTA, 100 mM NaCl, and 1% SDS, with a pH of 8.0). The solution was mixed using a vortex mixer before the addition of cold 5 M NaCl, 10 mg/mL Proteinase K, and 10 mg/mL RNase A. The solution was then incubated at 37 °C for 3 h. After incubation, DNA fragmentation was performed on 2% agarose gel at 60 volts for 3 h, and any fragments of DNA were visualized using a UV transilluminator.

#### 2.5.3. DNA Fragmentation Analysis using the TUNEL Assay

The TUNEL assay (terminal deoxynucleotidyl transferase and fluorescein-labeled dUTP, DNA Fragmentation Imaging Kit, Merck, Germany) was used as the standard method for detecting DNA fragmentation caused by apoptosis [31]. Briefly, the cancer cells, at a concentration of 2 × 10^5^ cells/mL, were exposed to the extract for 48 h. A total of three rounds of phosphate buffered saline (PBS, pH 7.4) washes were performed after cell harvesting. After that, the cells were fixed with 100 µL of 4% paraformaldehyde and allowed to incubate at room temperature for 10 min. Following the removal of the fixing solution, 100 µL of 0.1% Triton-X100 was added to the cells, and they were allowed to incubate at room temperature for 20 min. After that, the cells were cleaned by washing them twice in PBS (pH 7.4). After centrifugation, the cells were combined with 45 µL of the enzyme solution (terminal deoxynucleotidyl transferase, or TdT), and the mixture was then incubated at 37 °C in 5% CO_2_ for 1 h. Then, the cells were reacted with 150 µL of nuclei dye mixture solution (Hoechst 33342) before incubation in the dark at room temperature for 15 min. The reagent was separated out by centrifugation at 5000 g and 4 °C for 5 min. The pellets were resuspended with ProLongTM gold antifade mountant (Life Technologies, Camarillo, CA, USA) prior to the detection of the pellets containing fluorescent DNA fragments using an inverted fluorescence microscope (ECLIPSE Ts2R-FL, Nikon, Tokyo, Japan).

## 3. Results and Discussion

### 3.1. Phytochemical Characterization

**Figure 1:**
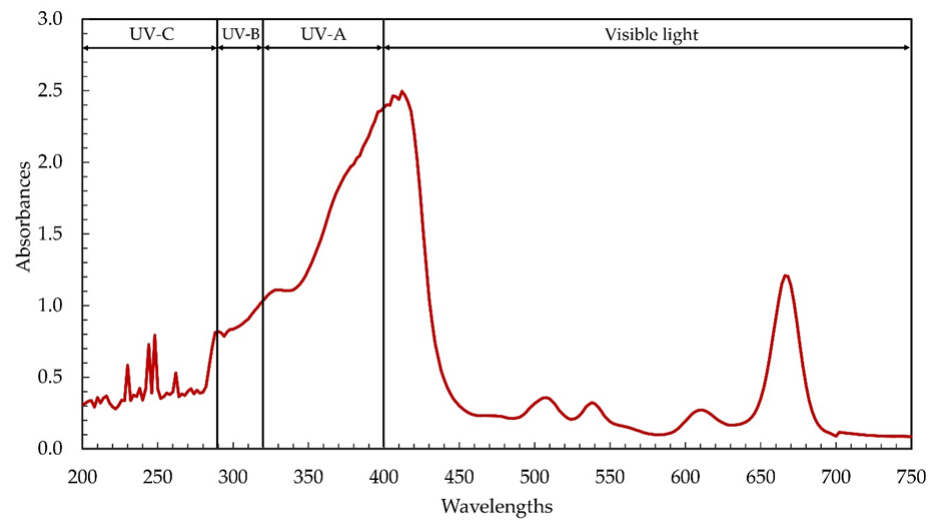
Ultraviolet–visible (UV–Vis) absorption spectra of the ethanolic extract from cyanobacteria, *Leptolyngbya* sp. KC45.

Microalgae, and cyanobacteria in particular, contain high concentrations of beneficial phytochemicals. In this study, the cyanobacterium *Leptolyngbya* sp. KC45 was assessed for phytochemical production through primary screening using the ultraviolet–visible (UV–Vis) spectrophotometric approach. Extracting phytochemicals required the use of a water-soluble solvent, strongly influencing extraction yields and bioactivities, and ethanol served this purpose well. Differences in phytochemical content and antioxidant activity between ethanol and other solvent extracts may be attributable to the strength and polarity of the solvent, which influence the solubility of bioactive chemicals [32]. It has been found that ethanol is an extremely effective solvent for the extraction of phytochemicals, resulting in a high phytochemical yield and excellent antioxidant activity [33]. Therefore, ethanol was used in this study to extract phytochemicals (termed ethanol extracts) from the biomass of the cyanobacterium *Leptolyngbya* sp. KC45. After the extraction step, the ethanolic extract yielded 48.54 g/kg-biomass (4.85% *w*/*w*), which is comparable to the extraction yields (0.1–10% *w*/*w*) reported for other types of cyanobacteria biomass, and there are several contributors to the phytochemical yields [5]. Figure 1 displays the UV–visible absorption patterns of phytochemicals from 200–700 nm, showing two primary absorption patterns at 280–450 nm and 640–690 nm, with additional, smaller absorption peaks at 220–270 nm, 490–525 nm, 525–550 nm, and 590–630 nm. Similarly, Joshi et al. [18] found that the crude methanolic extract of *Leptolyngbya* sp. exhibited strong absorption peaks between 290 and 520 nm and 550 and 700 nm. Peaks analogous to those seen in the ethanolic *Leptolyngbya* sp. extract in this study were also identified in the methanolic extract of *Leptolyngbya* sp. [19].

Previous discoveries demonstrating the complexity of phytochemical extracts, including the presence of phenolic compounds and pigments that absorb UV–visible light [5,6], corroborate our results. The ethanolic extract contained chlorophylls, carotenoids, phenolics, and flavonoids at 40.46 mg/g-extract, 1.58 mg/g-extract, 6.17 mg-GAE/g-extract, and 15.07 mg-QE/g-extract, respectively (Table 1). Among the chlorophylls detected, chlorophyll a and chlorophyll d were observed at 40.30 mg/g-extract and 0.15 mg/g-extract, respectively, while chlorophyll b and chlorophyll c were not detectable. Chlorophylls and carotenoids, two main types of cyanobacterial pigments extracted by solvent extraction, absorb light with wavelengths between 400 and 550 nm and 600 and 710 nm, respectively [5,6]. Maximum light absorption by chlorophyll a in most plants and algae was found, by Xu and Harvey [34], to occur around 680 nm, but maximum absorption by chlorophyll d, discovered in the cyanobacterium *Acaryochloris*, occurred between 460 and 706 nm [35]. The absorption peak of the ethanolic extract between 220 and 380 nm (Figure 1) determines the presence of phenolic compounds such as phenolic acids, flavonoids, flavonols, and anthraquinones [6]. Interestingly, the UV absorption peaks were seen in the ethanolic extract (Figure 1), with their respective peaks at 200–290 nm (UV-C), 290–320 nm (UV-B), and 320–400 nm (UV-A), respectively. The same kind of result was observed with the methanolic extract from *Leptolyngbya* sp. [18,19]. The absorption of UV also increased dramatically between the wavelengths of 280 and 400 nm, which correspond to UV-A, UV-B, and UV-C, suggesting that the ethanolic extract may be used in the formulation of sunscreen to shield the skin from harmful UV rays [6] and prevent premature aging [36].

Although phenolic molecules may substantially absorb UV light, some phenolic compounds, with their colorful nature, can lead to absorption characteristics in the visible light range [37]. To tentatively identify and characterize the phenolic compounds in the ethanolic extract, liquid chromatography–electrospray ionization–quadrupole time-of-flight–mass spectrometry (LC–ESI–QTOF/MS) was conducted by comparing their retention time (RT), mass error between the observed mass and the theoretical mass (<10 ppm), and MS data acquired under both negative and positive electron spray ionization modes (ESI^−^/ESI^+^). The data identification scores selected were over 80. As part of an LC–ESI–QTOF/MS study, positive mode ESI ionization was chosen due to its superior sensitivity over the negative mode. To achieve a positive ESI state during MS/MS operation, the [M + H]^+^ ion was selected using the MS scan mode. Therefore, in the case at hand, the observed compounds with good sensitivity based on the selected precursor ions were recorded, and all compounds suspected to be present in the ethanolic extract of *Leptolyngbya* sp. KC45 are listed in Table 2. Through LC–MS/MS, 38 phenolic compounds, including 9 phenolic acids, 20 flavonoids, 3 phenolic terpenes, 2 phloroglucinols, 2 phenolic glycosides, and 2 additional polyphenols, were found. 

A total of nine phenolic acids belonging to three different subclasses were tentatively identified in the extract (Table 2). Hydroxycinnamic acids were the dominant subgroup of phenolic acids, with seven compounds (Compounds 2, 3, 4, 5, 6, 7, and 8). Only one hydroxyphenylpropanoic acid compound (Compound 9) was tentatively identified, while another was hydroxybenzoic acid (Compound 1). Compounds 1, 2, 3, 4, 5, 6, 7, 8, and 9 were designated as vanillic acid 4-sulfate, cis-caffeoyl tartaric acid, m-coumaric acid, cinnamic acid, 2,5-dimethoxycinnamic acid, p-methoxycinnamic acid ethyl ester, cis-ferulic acid [arabinosyl-(1->3)-[glucosyl-(1->6)]-glucosyl] ester, 3,4,5-trimethoxycinnamic acid, and dihydrosinapic acid, respectively. Among phenolic acids, m-coumaric acid (Compound 3) and cinnamic acid (Compound 4) have already been identified in some cyanobacteria, such as *Nostoc* sp. and *Synechocystis* sp. [5,38,39].

A higher diversity of flavonoids was found among the phenolic compounds identified in the extract. A total of 20 flavonoids belonging to 5 subgroups were identified in this study. According to Table 2, the compounds 10–12, 13-18, 19–26, 27–28, and 29 were analyzed and classified as anthocyanins, flavanols, flavones, flavonols, and isoflavonoids, respectively. Malvidin 3-sophoroside 5-glucoside, curcumin monoglucoside, and malvidin 3-(6-coumaroylglucoside) 5-glucoside were compounds 10, 11, and 12, respectively. Compounds 13, 14, 15, 16, 17, and 18 were designated as (−)-epicatechin 7-*O*-glucuronide, 7-galloylcatechin, (±)-3′,4′-Methylenedioxy-5,7-dimethylepicatechin, epicatechin 3-*O*-(4-methylgallate), 3-methyl-epicatechin, and 4′,7-Di-*O*-methylcatechin, respectively. Compounds 19, 20, 21, 22, 23, 24, 25, and 26 were characterized as luteolin 4′-glucoside 7-galacturonide, 6-methoxyluteolin 7-glucuronide, 8-hydroxyluteolin 4′-methyl ether 7-(6‴-acetylallosyl) (1->2)(6″-acetylglucoside), 6-hydroxyluteolin 6,3′-dimethyl ether 7,4′-disulfate, 6-hydroxyluteolin 6,7- disulfate, luteolin 4′-methyl ether 7,3′-disulfate, 6-hydroxyluteolin 4′-methyl ester 7-rhamnosyl-(1->2)-(6″-acetylglucoside), and luteolin 4′-methyl ether 7-(4G-rhamnosylneohesperidoside), respectively. Compounds 27 and 28 were identified as quercetin 3-*O*-sulfate and quercetin 3-(2″- glucosylgalactoside) 7-glucoside, respectively. Compound 29 was detected as dalbergin. Among the flavonoids characterized, three compounds, including 7-galloylcatechin (Compound 14), epicatechin 3-*O*-(4-methylgallate) (Compound 16), and dalbergin (Compound 29), have already been reported in an unidentified cyanobacterial strain [40] and *Nostoc* sp. [5].

Nine other polyphenols were classified as phenolic glycosides (Compounds 30–31), methoxyphenols (Compound 32), phloroglucinols (Compounds 33–34), phenolic terpenes (Compounds 35–37), and lignan derivatives (Compound 38) (Table 2). Compounds 30 and 31 were tentatively identified as dihydrocaffeic acid 3-*O*-glucuronide and 5-(3′,5′-Dihydroxyphenyl)-gamma-valerolactone 3-*O*-glucuronide, respectively, which have already been observed in *Nostoc* sp. [5]. Compound 32 was curcumin I, and phloroglucinol and dihydrophloroglucinol were compounds 34 and 35, respectively. Phloroglucinol has been reported in some cyanobacteria, such as *Oscillatoria* sp., *Chroococcidiopsis* sp., *Leptolyngbya* sp., *Calothrix* sp., *Nostoc* sp., and *Phormidium* sp. [5,41], while dihydrophloroglucinol was found in *Nostoc* sp. [5]. Compounds 35, 36, and 37 were tentatively detected as carnosic acid, 11,12-Dimethylrosmanol, and 6,7-Dimethoxy-7-epirosmanol, respectively, which have already been observed in *Nostoc* sp. [5]. Compound 38 was identified as 2′-hydroxyenterolactone.

Table 3 shows the comparison of identified compounds with previous studies [42,43,44,45,46,47,48,49,50,51,52,53,54,55,56,57,58,59,60,61,62,63,64,65,66,67,68,69,70,71,72,73,74,75,76,77,78,79,80,81,82,83,84,85,86,87,88,89,90,91,92,93,94,95,96,97,98,99,100] on bioactivities. It was found that there have been no investigations on the bioactivities of compounds 1, 5, 7, 12–16, 18, 21–25, or 28. Interestingly, compounds 2–4, 6, 8–11, 17, 19, 20, 26–27, and 29–38 were already known to have antioxidant properties, confirming that the availability of the compounds in *Leptolyngbya* ethanolic extract might play an important role in stabilizing harmful oxidants. Fourteen of the thirty-eight identified phenolic compounds were found to be enzyme inhibitors. These enzyme-related disease inhibitors include compounds 2–4, 6, 8, 9, 14, 19, 26, 32, 33, and 35–37. More importantly, half of all identified phenolic compounds, including compounds 2–4, 6, 8–11, 13, 14, 27, 29, and 32–38, were characterized as potential cancer inhibitors, indicating that these compounds might contribute to the anticancer activity of *Leptolyngbya* ethanolic extract. This shows that *Leptolyngbya* sp. ethanolic extract could be used in the pharmaceutical, nutraceutical, and functional food industries. However, quantitative studies on identified phenolic profiling should be examined in further studies by comparing with standard compounds in order to expand the current understanding of the phytochemical characterization. Overall, the phytochemical profile of *Leptolyngbya* sp. ethanolic extract indicates the presence of potentially beneficial pigments and phenolic compounds. Thus, the application of *Leptolyngbya* sp. ethanolic extract might have a sizeable bearing on the process of industrializing and commercializing the bioactive ingredients. 

### 3.2. Bioactivity Potentials

#### 3.2.1. Antioxidant Activity

**Figure 2:**
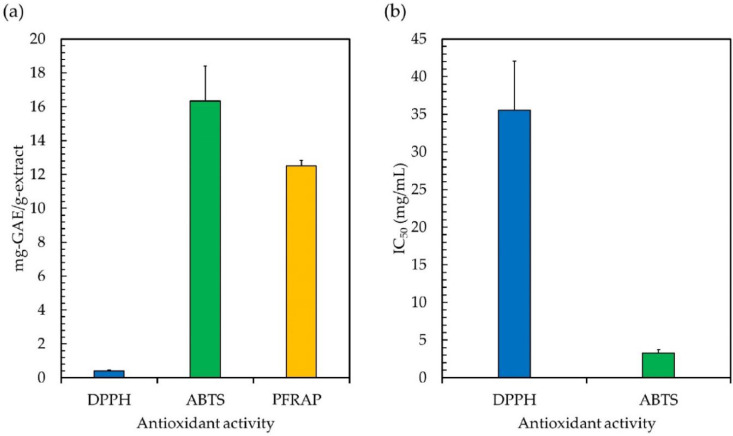
The gallic acid equivalent antioxidant capacity (mg-GAE/g-extract) (**a**) and the half maximal inhibitory concentration (IC_50_; mg/mL) on 2,2-diphenyl-1-picrylhydrazyl (DPPH), 2,2′-azino-bis (3-ethylbenzthiazoline-6-sulfonic acid) (ABTS), and potassium-ferricyanide-reducing antioxidant power (PFRAP) (**b**) of the ethanolic extract from cyanobacteria, *Leptolyngbya* sp. KC45.

Since free radicals may cause a lot of damage to foods and biological systems, antioxidant properties are crucial [6]. The three most prevalent types of antioxidant tests, i.e., 2,2-diphenyl-1-picrylhydrazyl (DPPH), 2,2′-azino-bis (3-ethylbenzthiazoline-6- sulfonic acid) (ABTS), and potassium-ferricyanide-reducing antioxidant power (PFRAP), were employed to determine the levels of antioxidant activity in the ethanolic extract. DPPH is a persistent free radical that is purple in color. It has been extensively used to test the antioxidant capacity of various substances, and it displays a characteristic absorption band at 517 nm [24]. This technique relies on the creation of the non-radical form DPPH-H, which results from the reduction of the stable free radical DPPH in the presence of a hydrogen-donating antioxidant [24]. As shown in Figure 2a,b, the DPPH radical scavenging activity of the ethanolic extract had an IC_50_ of 35.51 mg/mL, with the gallic acid equivalent antioxidant capacity of 0.40 mg-GAE/g-extract, indicating that *Leptolyngbya* phytochemicals include efficient hydrogen-donating molecules that will convert the DPPH free radicals into a more stable state [27]. This demonstrates that the phytochemicals derived from *Leptolyngbya* sp. KC45 might possibly be employed as a DPPH inhibitor to protect cells from oxidative stress by scavenging DPPH free radicals. It was discovered that chlorophylls [6], carotenoids [101], and phenolics [5] could all play a significant role in the process of scavenging DPPH free radicals. Similarly, the phytochemicals obtained from plants and algae with different solvent extraction methods, such as the methanolic extract of edible macroalgae [1], the methanol extract of *Clausena excavata*, *Clausena harmandiana*, and *Murraya koenigii* [30], the methanolic extracts of *Leptolyngbya* sp. SI-SM [41], the methanolic and ethanolic extracts of *Leptolyngbya* sp. LMECYA 173 [8], and the diethyl ether extracts of *Leptolyngbya* sp. DEEL-3 [15], can effectively neutralize the DPPH free radical to a more stable form by providing IC_50_ values of 0.08–50.0 mg/mL. 

To test the antioxidant potential of the ethanolic extract based on hydrogen-donating antioxidants against nitrogen radicals, the ABTS radical scavenging activity assay was employed in this study. In this assay, the reducing power of an antioxidant is measured by its ability to neutralize a colored stable ABTS free radical. By reducing the absorbance of samples at 734 nm, spectrophotometry can quantify the decolorization of an ABTS solution caused by the hydrogen provided by antioxidant components in the extract reacting with ABTS radicals [19]. This capacity is used to define an antioxidant’s level of antioxidant activity [25]. According to Figure 2a,b, the ethanolic extract showed antioxidant potential by scavenging ABTS with an IC_50_ value of 3.31 mg/mL (16.33 mg-GAE/g-extract), which is in good agreement with the previous publications reported by Ijaz and Hasnain [41], Ghareeb et al. [44], Anas et al. [102], and Mali et al. [103]. They found that the phytochemicals of various plants and algae extracted with different solvents, such as methanolic extracts, ethanolic extracts, and diethyl ether extracts, gave IC_50_ values in the range of 0.07–8.00 mg/mL. This demonstrates that the ABTS free radicals are stabilized by accepting a hydrogen ion from the phytochemicals. It was shown that phenolic compounds, especially phenolic acids and flavonoids, and pigments, such as chlorophylls and carotenoids, contribute significantly to ABTS radical scavenging action [103]. As a result, the *Leptolyngbya* phytochemicals not only remove free radicals by scavenging DPPH, but they also stabilize ABTS free radicals, which implies they have the potential to be employed as oxidant inhibitors to protect cells from the damaging effects of oxidative stress.

Aside from the mechanism of scavenging harmful free radical reactions, the ability of the ethanolic extract to reduce Fe^3+^ ions to the blue Fe^2+^ ion complex was measured using the PFRAP assay. The PFRAP test is a standard approach that was created to measure the electron-donating capacity of bioactive substances, which may be reflective of their antioxidant potency [27]. In the presence of antioxidants, the reduction of the Fe^3+^ ferricyanide complex to the Fe^2+^ complex results in the creation of the intense Perl’s Prussian blue complex, which possesses a high absorbance at 700 nm. If the absorbance of the reaction solution at 700 nm is higher, this implies that it has a greater capacity to reduce ferric ions [19]. The ethanolic extract exhibited PFRAP activity, with a value of 12.51 mg-GAE/g-extract (Figure 2a), which is in line with previously reported phytochemicals from the methanol extract of *Clausena excavata*, *Clausena harmandiana*, and *Murraya koenigii* [30], the hexane and ethyl acetate extracts of *Leptolyngbya* sp. [102], and the methanolic extract of *Leptolyngbya* sp. [19], providing PFRAP activity of 0.1–50 mg-GAE/g-extract. In the biological system, Fe^3+^ ions are among the most powerful pro-oxidants, and their combination with H_2_O_2_ results in the generation of harmful OH radicals. An abnormal accumulation of metal ions in the cellular system can also result in a variety of different abnormalities [104]. Therefore, the ethanolic extract with the ability to reduce the ferric ion obtained in this study could prevent the generation of OH radicals, resulting in the absence of oxidative damage. In the literature, this extract, which is abundant in pigments and phenolic compounds, has demonstrated outstanding PFRAP activity, and has been found to be an effective scavenger of reactive species such as OH radicals, oxygen radicals, and reactive oxygen species [5,6]. Overall, the ethanolic extract of *Leptolyngbya* sp. KC45 displayed significant antioxidant capacity, which consisted of one or two radical scavenging mechanisms. This indicates that the *Leptolyngbya* sp. KC45 extract might potentially represent a promising antioxidative functional component.

#### 3.2.2. Tyrosinase Inhibitory Activity

**Figure 3:**
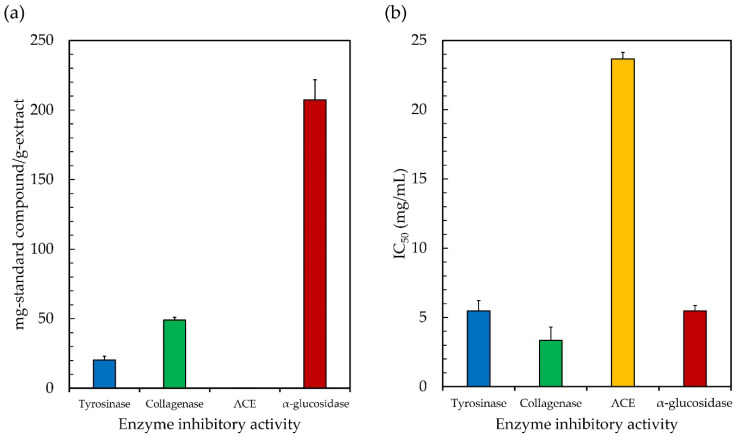
The half maximal inhibitory concentration (IC_50_) (**a**) and the equivalent standard compound capacity (**b**) of the ethanolic extract from cyanobacteria, *Leptolyngbya* sp. KC45 on enzyme inhibitory activity.

Melanin is the pigment that is responsible for the skin, and there is a possibility that persistent exposure to the sun might cause an excessive amount of melanin production, which is typically correlated with skin problems such as hyperpigmentation, age spots, and freckles [27]. The production of melanin requires the catalytic hydroxylation of L-tyrosine to L-3,4-dihydroxyphenylalanine (L-DOPA), and the subsequent oxidation of L-DOPA to dopaquinone, both of which are catalyzed by the copper-containing enzyme tyrosinase [26]. It is possible to regulate the formation of melanin by limiting the activity of the tyrosinase enzyme. Jesumani et al. [36] proposed that the suppression of tyrosinase activity is a relevant factor in determining the skin-whitening effect of a substance. Thus, it is crucial to investigate the natural substances that have been shown to have strong tyrosinase inhibitory activity. In this study, the ethanolic extract of *Leptolyngbya* sp. KC45 was tested for its ability to inhibit tyrosinase, using L-DOPA as a substrate, to determine whether or not it should be pursued as a potential source of antiaging ingredients for use in cosmetics. Based on Figure 3, the ethanolic extract was found to have positive tyrosinase inhibitory activity, with an IC_50_ value of 5.48 mg/mL (20.37 mg-KE/g-extract), which is similar to the various solvent extracts from different natural sources (IC_50_ of 0.02–7.4 mg/mL), such as microalgae [105], cyanobacteria [106], and plants [107]; therefore, it has the potential to be used in the creation of a tyrosinase inhibitor for skincare products. The presence of anti-tyrosinase activity was shown to be associated with the presence of pigments and phenolic compounds in the extract, which is consistent with the findings reported by Jesumani et al. [36] and Klomsakul and Chalopagorn [108]. According to Ruangrit et al. [26], the enzyme may undergo steric obstruction or conformational change as a result of the extract, which contains effective hydroxyl groups and has the potential to form hydrogen bonds at a location on the tyrosinase enzyme. Similarly, phenolic hydroxyl penetrates the hydrophobic cavity of tyrosinase, causing a conformational shift in the enzyme that, in turn, has an effect on its catalytic activity [109]. Phenolic substances, and phenolic acids in particular, have phenol rings that include at least one hydroxyl group, which allows them to suppress the activity of tyrosinase enzymes [110]. The inhibition of tyrosinase activity will be strongly impacted by both the quantity of phenolic hydroxyl groups present in the flavonoids and their locations within the molecule [109]. Based on these results, it seems that the ethanolic extract of *Leptolyngbya* sp. KC45 has the potential to inhibit tyrosinase, scavenge free radicals, and absorb UV light, all of which are desirable properties in a skin-whitening formulation.

#### 3.2.3. Collagenase Inhibitory Activity

Since collagenase is the enzyme that is capable of degrading collagen, which is a major component of the extracellular matrix that plays key functions in skin tightening and skin resilience, inhibiting this enzyme would lead to a delay in the aging process associated with the skin [111,112]. Collagen fibers have usually been thought of as the main fibrillar component of the skin’s dermis layer, a layer of connective tissue between the outermost skin layer (the epidermis) and the deepest skin layer (the subcutaneous fat layer). However, when free radicals build up in the skin following exposure to photoaging stimuli, the collagenase enzyme might be activated indirectly, contributing to the accelerated aging of the skin, characterized by the emergence of wrinkles, freckles, sallowness, and/or deep furrows, or by severe atrophy, laxity, and/or leathery texture of the skin [113]. More importantly, the excessive production of the collagenase enzyme was shown to cause dry skin in adults older than 60 years. This can lead to severe xerosis, which is marked by skin inflammation, itching, and fissured and cracked skin [112]. As a result, the inhibition of collagenase, along with the stabilization of damaging free radicals, is crucial for the prevention of cell damage. In this study, the inhibition effects of the ethanolic extract of *Leptolyngbya* sp. KC45 on collagenase enzyme activity were investigated, and are shown in Figure 3. The ethanolic extract can inhibit the collagenase enzyme at IC_50_ rates of 3.36 mg/mL (49.12 mg-EGCGE/g-extract), which are in line with the extracts (IC_50_ = 0.01–6 mg/mL) from white tea [28], glutinous rice husk [111], *Turbinaria decurrens* Bory [114], and *Citrus unshiu* orange [115]. The phytochemical extract’s pigments [114] and phenolic components [115] have collagenase inhibitory action. According to Mechqoq et al. [116], as reported in the literature, phenolic compounds, which include phenolic acids and flavonoids, have a high affinity for collagenase, and have the ability to interact with the active sites of collagenase by forming hydrogen bond interactions with those sites in order to block the enzyme. Therefore, it is important to highlight that the ethanolic extract of *Leptolyngbya* sp. KC45 has the virtues of inhibiting tyrosinase and collagenase, and has significant antioxidant activities, making it a potential bioactive component in the cosmeceutical sector.

#### 3.2.4. Angiotensin-Converting Enzyme (ACE) Inhibitory Activity

The most important contributor to the development of cardiovascular disease is high blood pressure (hypertension). Hypertension is one of the most common health problems in the world. It is linked to many other health problems, such as heart disease, chronic kidney failure, aneurysm, and stroke [117]. Blocking the angiotensin-I-converting enzyme (ACE) is an important part of treating hypertension because of its role in blood pressure regulation. In the human body, ACE is the major enzyme in the renin–angiotensin system (RAS) pathway. It is responsible for the conversion of the inactive form of angiotensin I (Ang I) into the strong vasoconstrictor angiotensin II (Ang II; highly active form) [118]. ACE is also responsible for the destruction of bradykinin, which is a vasodilator. Bradykininogen is the substrate from which active hypertensive bradykinin is derived; afterwards, kinase II catalyzes the degradation of bradykinin into inactive components [119]. As a result, the inhibition of ACE has emerged as a viable contemporary therapeutic strategy for the treatment of hypertension. In this study, the ACE-inhibiting potential of the ethanolic extract of *Leptolyngbya* sp. KC45 was evaluated. The ethanolic extract showed potent ACE inhibition, with IC_50_ values of 23.67 mg/mL (0.15 mg-EE/g-extract) (Figure 3); however, these values were still lower than those seen with enalapril (0.0035 mg/mL) as a commercial standard drug. Although the ethanolic extract has lower ACE inhibitory activity than enalapril, it has the potential to be used as a substitute for ACE inhibitors in order to protect patients from the potentially harmful side effects of enalapril and other medicines that are commercially related [29]. There was a possibility that some of the pigments and phenolic chemicals in the ethanolic extract acted as ACE inhibitors or as molecules that were cardioprotective. Similarly, both Liu et al. [120] and Sukandar et al. [121] noted that ACE inhibitory effects were associated with phenolic components such as phenolic acids and flavonoids identified in plant ethanolic extracts, which might decrease the production of Ang II and reduce the degradation of bradykinin [118]. According to Ali et al. [117], the ability of phenolic acids to suppress ACE activity was shown to be affected by whether or not particular functional groups (hydroxyl, carboxyl, and ketone) were present in the extract. In addition, several hydrogen bonds and hydrophobic interactions between phenolic chemicals and ACE’s catalytic residues were shown to be crucial in inhibiting the enzyme’s catalytic activity [122]. Until now, a lot of effort has been put into exploring the natural product universe for ACE inhibitors, in the hopes of discovering ones with more favorable pharmacological profiles and fewer adverse effects. The obtained ethanolic extract of *Leptolyngbya* sp. KC45 in this study would thus provide potential medicinal and economic advantages as a functional component.

#### 3.2.5. α-Glucosidase Inhibitory Activity

α-Glucosidase is an important enzyme that plays a role in the digestive process of carbohydrates that occur in the human body. This enzyme plays a role in the release of glucose from disaccharides or complex carbohydrates, hence contributing to an increase in postprandial blood glucose levels [123]. Inhibition of this enzyme is one of the therapeutic options that may be used in the treatment of diabetic complications. It is possible to reduce postprandial hyperglycemia by blocking α-glucosidase, which results in a slowing of both the digestion of carbohydrates and the absorption of glucose [124]. Several synthetic α-glucosidase inhibitors, including metformin and acarbose, are commercially available for the treatment of diabetes mellitus. The use of these synthetic drugs, on the other hand, has been linked to a number of negative side effects in diabetes patients, including abdominal distention, flatulence, meteorism, and diarrhea [125]. Given the high cost and frequent adverse effects of many antidiabetic drugs, natural ingredients have become a popular alternative for obtaining hypoglycemic agents. In this study, the ethanolic extract of *Leptolyngbya* sp. KC45 inhibited the α-glucosidase enzyme with a IC_50_ value of 5.47 mg/mL (207.24 mg-AE/g-extract) (Figure 3), which is in line with the extracts (IC_50_ = 0.01–50 mg/mL) from black legumes [123], shmar (*Arbutus pavarii* Pamp) [124], guarana [125], and algae [126]. The high inhibitory activity against the α-glucosidase enzyme may be due to the high concentration and variety of phenolic chemicals found in the extract. Similarly, along with their powerful antioxidant action, phenolic-rich samples have been shown to have a high potential for suppressing the activity of the α-glucosidase enzyme [124]. In addition, several studies have demonstrated that phytochemicals from plants and algae might be a rich natural supply of phenolic and flavonoid compounds, which are well-known for their significant hypoglycemic potential [126,127]. It is important to point out that the ethanolic extract obtained from *Leptolyngbya* sp. KC45 has a substantial proportion of substances that inhibit α-glucosidase. However, the α-glucosidase inhibitory activity of the extract was still lower than that of acarbose as a commercial synthetic drug (IC_50_ = 1.13 mg/mL). Hence, we propose that combining the use of ethanolic extract with a low dosage of synthetic drugs could be one of the alternative possibilities for treating hyperglycemia with minimal side effects. In the literature, Proenca et al. [128], Ahn et al. [129], and Etsassala et al. [130] found that phenolic and flavonoid phytochemicals can limit α-glucosidase activity by inserting themselves into the active site of the enzyme and interacting with it in various ways, including by traditional hydrogen bonding and carbon–hydrogen bonding. It can be inferred that the ethanolic extract, which exhibits α-glucosidase inhibitory activity, obtained in this study might block the substrate-binding active site of the enzyme, thereby preventing the oligosaccharides from accessing the active site. Thus, the ethanolic extract of *Leptolyngbya* sp. KC45 might be beneficial for glucose-related diseases.

### 3.3. Cytotoxicity

**Figure 4:**
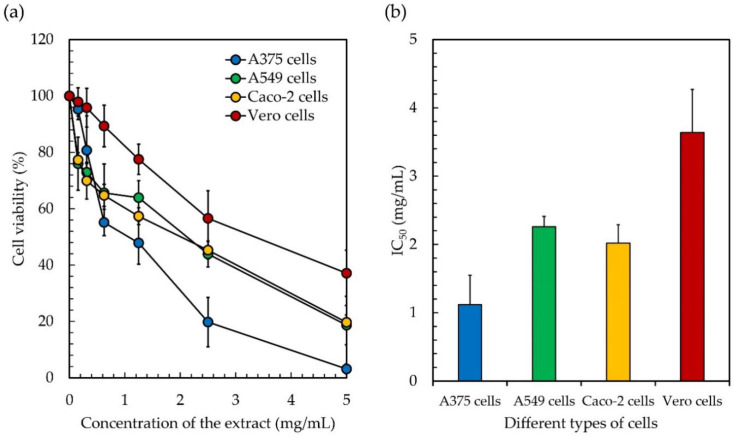
The cytotoxicity of skin cancer A375 cells, lung cancer A549 cells, and colon cancer Caco-2 cells after treatment with the ethanolic extract from cyanobacteria, *Leptolyngbya* sp. KC45 when compared with control cells, i.e., normal Vero cells, for 48 h. Cell viability was measured using the 3-(4,5-Dimethylthiazol-2-yl)-2,5-diphenyltetrazolium bromide reduction (MTT) assay (**a**) and calculated for comparisons with the control cells (**b**).

In addition to the antioxidant and enzyme inhibitory abilities obtained from the studies on *Leptolyngbya* sp. KC45 ethanolic extract, the cytotoxicity test (or antiproliferative activity) of the ethanolic extract by 3-(4,5-Dimethylthiazol-2-yl)-2,5-diphenyltetrazolium bromide reduction (MTT) assay was carefully conducted against three types of human cancer cell, namely, skin cancer A375 cells, lung cancer A549 cells, and colon cancer Caco-2 cells, compared to normal Vero cells. The results reveal that the cell viabilities of cancer cells and normal cells decline linearly with increasing concentrations of the extract (Figure 4). After 48 h of treatment, the ethanolic extract exhibited antiproliferative activity on A375 cells with an IC_50_ of 1.11 mg/mL, A549 cells with an IC_50_ of 2.26 mg/mL, Caco-2 cells with an IC_50_ of 2.02 mg/mL, and Vero cells with an IC_50_ of 3.64 mg/mL. Since a low IC_50_ value means that the extract is effective at low concentrations, the ethanolic extract appeared to have a significant amount of anticancer action, indicating that all three cancer cells, but not normal cells, exhibited a moderate to very significant antiproliferative impact after being exposed to the extract. However, there are no standard IC_50_ values or ranges for comparison. In general, the extract should have exhibited a lower IC_50_ when applied to cancer cells compared to normal cells. Similarly, Trabelsi et al. [131] suggested that the extract, which is more sensitive to cancer cells but not hazardous to normal cells, might be used as a model for the development of a program to treat cancer. In the literature, the selectivity index (SI) indicates the selectivity of a given compound between normal and cancer cells [132]. The SI was calculated as the average of the IC_50_ value in the normal cell line divided by the IC_50_ value in the cancer cell line obtained in each independent experiment [133]. The SI of the ethanolic extract ranged from 1.6 to 3.2, indicating that the extract was more effective against cancer cells than toxic to normal cells. However, an SI value ≥10 was assumed to belong to a selected potential sample that can be further investigated [132]. Weerapreeyakul et al. [134] proposed a lower SI value (≥3) for classifying a prospective anticancer sample. A high SI value at ≥2 suggests a promising alternative or complementary treatment for cancer patients [135]. While Krzywik et al. [136] suggested that if the extract has a favorable SI greater than 1.0, this means that it is more effective against cancer cells than it is hazardous to normal cells, indicating the potential use of the extract as an inhibitor for cancer treatment. Although the ethanolic extract has a low SI, it has the potential to be used as a substitute for cancer treatment in order to protect patients from the potentially harmful side effects of commercial drugs [137]. Therefore, it is interesting to note that in this study, cancer cells, in comparison to normal cells, exhibited a higher level of sensitivity, which might lead to the development of effective anticancer applications that have a lower level of toxicity for the body. 

In line with our results, cancer cells of the types HeLa, MCF-7, Hep3B, A549, C6, and HT29 were found to be sensitive to the cytotoxicity of a methanolic extract of four cyanobacteria (*Chroococus minutus*, *Geitlerinema carotinosum*, *Nostoc linckia*, and *Anabaena oryzae*) rich in pigments and phenolics [138]. Our findings are consistent with those of Kim et al. [139], Mesas et al. [140], and N’guessan et al. [141], who all found that ethanolic extracts of medicinal plants exhibited comparable cytotoxicity toward human cancer cells (T84, HT1080, MCF-7, MDA-MB231, and HCT-15). They also proposed that the presence of chlorophyll, carotenoids, and phenolics in the extract could be associated with the antioxidant and anticancer effects that these compounds possess. This indicates that the anticancer properties seen in the ethanolic extract are attributed to the presence of chlorophylls, carotenoids, phenolics, and flavonoids, and that the phytochemical extract from *Leptolyngbya* sp. KC45 has potential as a bioactive component with applications in the nutraceutical, cosmetic, and pharmaceutical sectors. However, before the extract can be used as a therapeutic agent, more research is needed regarding its effects on animal models, as well as clinical evaluations of the extract’s efficacy.

**Figure 5:**
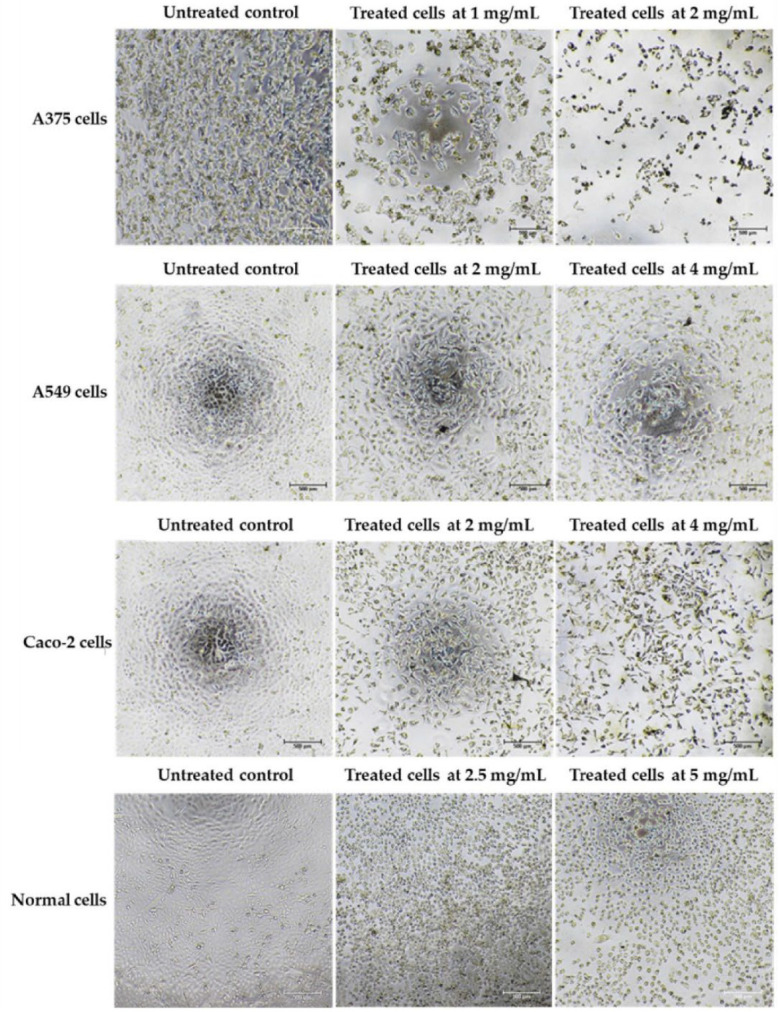
Morphology of skin cancer A375 cells, lung cancer A549 cells, colon cancer Caco-2 cells, and normal Vero cells after treatment with the ethanolic extract from cyanobacteria, *Leptolyngbya* sp. KC45, when compared with control (untreated) cells for 48 h.

**Figure 6:**
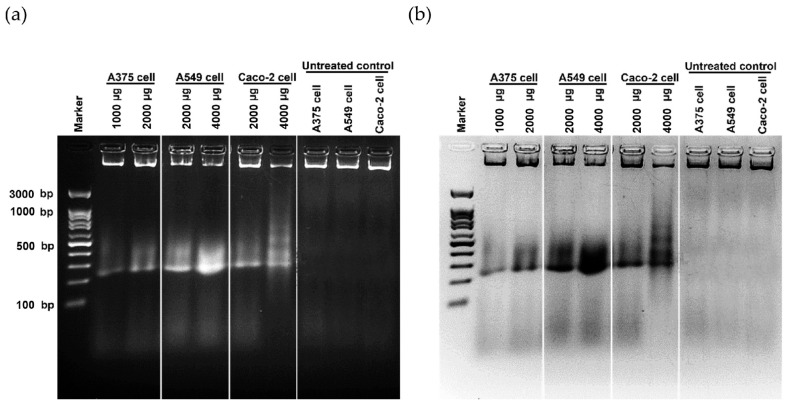
Deoxyribonucleic acid (DNA) fragmentation of skin cancer A375 cells, lung cancer A549 cells, and colon cancer Caco-2 cells after treatment with the ethanolic extract from cyanobacteria, *Leptolyngbye* sp. KC45, for 48 h when compared with control cells, i.e., normal Vero cells, by agarose gel electrophoresis (**a**) and inverting (black/white) the agarose gel (**b**).

Cancer can be defined in large part by the resultant uncontrolled cell proliferation and the limitation of apoptosis in the affected cells. Traditional cancer therapies have focused on inhibiting cell division and killing cancerous cells through a process called apoptosis [142]. In this study, the morphological features of apoptosis were studied to understand how the ethanolic extract of *Leptolyngbya* sp. KC45 could promote apoptosis. After treating cancer cells with the extract at different concentrations for 48 h, treated cells lost their ability to adhere to one another, shrank, and rounded off when compared with untreated cells (Figure 5). The increasing concentration of the extract also proved the presence of apoptosis-related phenomena in the apoptotic cells, such as cell shrinkage, membrane blebbing condensation, margination of nuclear chromatin, apoptotic bodies, and engulfment by neighboring cells (Figure 5), which corroborates the findings of Kaewkod et al. [31]. In addition, the activation of deoxyribonucleic acid (DNA) fragmentation was studied in order to validate the apoptosis process. DNA fragmentation is one of the features that can be used to identify apoptotic cell death [143]. Through the agarose gel electrophoresis analysis (Figure 6), DNA fragments between 200 and 1000 bp were found in cancer cells treated with the ethanolic extract, and strong DNA bands were also observed with increasing concentrations of the extract, suggesting that this extract can trigger apoptosis-induced DNA fragmentation in all three cancer cells.

**Figure 7:**
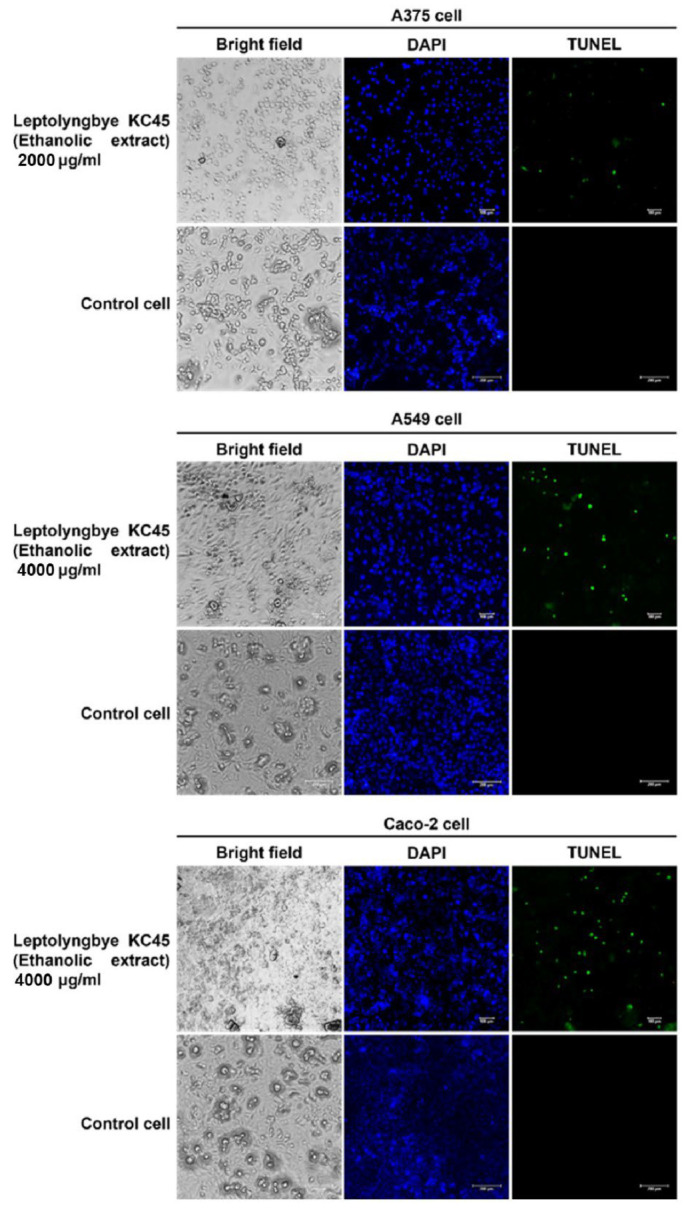
Terminal deoxynucleotidyl transferase dUTP nick end labeling (TUNEL) assay of skin cancer A375 cells, lung cancer A549 cells, and colon cancer Caco-2 cells after treatment with the ethanolic extract from cyanobacteria, *Leptolyngbya* sp. KC45, when compared with control cells, i.e., normal Vero cells, for 48 h. The cells were stained with 4′,6-diamidino-2-phenylindole (DAPI) and TUNEL, and observed under a fluorescent microscope.

The terminal deoxynucleotidyl transferase dUTP nick end labeling (TUNEL) assay also serves as a confirmation test for the detection of apoptosis DNA fragmentation [3]. In this study, the impact of the ethanolic extract on the DNA damage of three different types of cancer cells was discovered by labeling the cancer cells with 4′,6-diamidino-2-phenylindole (DAPI) and TUNEL fluorescent dyes after 48 h of treatment with the extract. Staining with DAPI, which emits blue fluorescence, can show whether a cell is alive or dead by revealing its nucleus. Only DNA deterioration may be detected by TUNEL labeling, which uses terminal deoxynucleotidyl transferase (TdT) to integrate labeled dUTP into free hydroxyl termini formed after fragmentation of genomic DNA [144]. According to Figure 7, the results show that after being treated with the extract, the nuclei of all three types of cancer cells exhibited green fluorescence from TUNEL labeling; on the other hand, the nuclei of untreated cancer cells did not exhibit this fluorescence, indicating that all three types of cancer cells fragmented their DNA similarly when tested using agarose gel electrophoresis, confirming the effects of the ethanolic extract. According to the findings of this investigation, the ethanolic extract of *Leptolyngbya* sp. KC45 might potentially be used in the treatment of cancer in the future. However, further studies on the analysis of the expression of genes related to apoptosis should be investigated for a better understanding of the gene expression of molecules regulating apoptotic pathways in cancer cells treated with the extract.

Following several stages employed in this study, the ethanolic extract from *Leptolyngbya* sp. KC45 might potentially be used in the future as an alternative to scavenging harmful free radicals, and as an enzyme-related disease inhibitor, as well as a cancer therapeutic agent. However, this study was only carried out as an in vitro investigation. For future study, an in vivo assay is needed to determine how the body will respond to the extract, since in vivo studies investigate the actual effect on an organism, whether it be a laboratory animal or a human. Clinical trials or medical studies may be performed either in vivo or in vitro. These methods are comparable in that they are both carried out with the intention of making breakthroughs in the study and treatment of illness and disease, in addition to gaining an understanding of “health” and the normal biological functioning of the human body. Therefore, it is important to note that both in vitro and in vivo studies should be further conducted to establish what might result when the extract is used in real applications.

## 4. Conclusions

According to our findings, the ethanolic extract obtained from *Leptolyngbya* sp. KC45 contained significant amounts of useful phytochemicals, which have functional qualities and bioactive characteristics. The characterization of the phytochemicals and assessment of their bioactivity potentials point towards the possibility of applications in the pharmaceutical, nutraceutical, and functional food sectors. Due to the extract’s antioxidant and enzyme inhibitory capabilities, there is the possibility that it might be used in the future as an alternative to scavenging free radicals and as an enzyme-related disease inhibitor. The extract also had a potentially detrimental impact on cancer cells through DNA fragmentation, validating the innate apoptosis pathways. Our results suggest that the phytochemically rich, multifunctionally active ethanolic extracts of *Leptolyngbya* sp. KC45 have significant biotechnological potential.

## Figures and Tables

**Table 1:** Phytochemical composition of the ethanolic extract from cyanobacterium *Leptolyngbya* sp. KC45.

Phytochemicals	Concentration
Chlorophyll a (mg/g-extract)	40.30 ± 0.21
Chlorophyll b (mg/g-extract)	Not detectable
Chlorophyll c (mg/g-extract)	Not detectable
Chlorophyll d (mg/g-extract)	0.15 ± 0.05
Total chlorophylls (mg/g-extract)	40.46 ± 0.08
Carotenoids (mg/g-extract)	1.58 ± 0.03
Total phenolics (mg-GAE/g-extract)	6.17 ± 2.56
Total flavonoids (mg-QE/g-extract)	15.07 ± 1.17

**Table 2:** Liquid chromatography–electrospray ionization–quadrupole time-of-flight–mass spectrometry (LC–ESI–QTOF–MS/MS) polyphenol profiling in the ethanolic extract of *Leptolyngbya* sp. KC45.

No.	Proposed Compounds	Retention Time (min)	Mode of Ionization	Referenced Mass	Observed Mass	Mass Error (ppm)	Cyanobacteria
**Phenolic acid**							
*Hydroxybenzoic acids*							
1	Vanillic acid 4-sulfate	1.294	[M + H]^+^	247.9991	247.9995	1.85	NA
*Hydroxycinnamic acids*							
2	Cis-Caffeoyl tartaric acid	1.478	[M + H]^+^	312.0481	312.0500	5.99	NA
3	m-Coumaric acid	1.561	[M + H]^+^	164.0473	164.0469	−2.56	*Nostoc* sp. [5], *Synechocystis* sp. [38]
4	Cinnamic acid	4.107	[M + H]^+^	148.0524	148.0522	−1.51	*Synechocystis* sp. [39]
5	2,5-Dimethoxycinnamic acid	13.578	[M + H]^+^	208.0736	208.0717	−8.99	NA
6	p-Methoxycinnamic acid ethyl ester	43.338	[M + H]^+^	206.0943	206.0930	−6.22	NA
7	cis-Ferulic acid [arabinosyl-(1->3)-[glucosyl-(1->6)]-glucosyl] ester	80.355	[M + H]^+^	650.2058	650.2104	7.11	NA
8	3,4,5-Trimethoxycinnamic acid	82.285	[M + H]^+^	238.0841	238.0844	1.37	NA
*Hydroxyphenylpropanoic acids*							
9	Dihydrosinapic acid	1.594	[M + H]^+^	226.0841	226.0860	8.41	NA
**Flavonoids**							
*Anthocyanins*							
10	Malvidin 3-sophoroside 5-glucoside	1.411	[M + H]^+^	817.2402	817.2369	−4.06	NA
11	Curcumin monoglucoside	1.444	[M + H]^+^	530.1788	530.1825	7.02	NA
12	Malvidin 3-(6-coumaroylglucoside) 5-glucoside	79.872	[M + H]^+^	801.2242	801.2163	−9.82	NA
*Flavanols*							
13	(−)-Epicatechin 7-*O*-glucuronide	1.444	[M + H]^+^	466.1111	466.1065	−9.92	NA
14	7-Galloylcatechin	1.494	[M + H]^+^	442.0900	442.0857	−9.83	Unidentified cyanobacteria [40]
15	(±)-3′,4′-Methylenedioxy-5,7-dimethylepicatechin	1.511	[M + H]^+^	330.1103	330.1105	0.41	NA
16	Epicatechin 3-*O*-(4-methylgallate)	1.627	[M + H]^+^	456.1056	456.1041	−3.30	*Nostoc* sp. [5]
17	3-methyl-epicatechin	85.814	[M + H]^+^	304.0947	304.0958	3.69	NA
18	4′,7-Di-*O*-methylcatechin	86.163	[M + H]^+^	318.1103	318.1125	6.70	NA
*Flavones*							
19	Luteolin 4′-glucoside 7-galacturonide	1.511	[M + H]^+^	624.1326	624.127	−9.00	NA
20	6-Methoxyluteolin 7-glucuronide	1.527	[M + H]^+^	492.0904	492.0861	−8.69	NA
21	8-Hydroxyluteolin 4′-methyl ether 7-(6‴-acetylallosyl)(1->2)(6″-acetylglucoside)	1.644	[M + H]^+^	724.1851	724.1915	8.89	NA
22	6-Hydroxyluteolin 6,3′-dimethyl ether 7,4′-disulfate	1.694	[M + H]^+^	489.9876	489.9897	4.24	NA
23	6-Hydroxyluteolin 6,7- disulfate	49.28	[M + H]^+^	461.9563	461.9526	−7.88	NA
24	Luteolin 4′-methyl ether 7,3′-disulfate	56.137	[M + H]^+^	459.9770	459.9779	1.93	NA
25	6-Hydroxyluteolin 4′-methyl eter 7-rhamnosyl-(1->2)-(6″-acetylglucoside)	79.988	[M + H]^+^	666.1796	666.1801	0.70	NA
26	Luteolin 4′-methyl ether 7-(4G-rhamnosylneohesperidoside)	80.321	[M + H]^+^	754.2320	754.2272	−6.36	NA
*Flavonols*							
27	Quercetin 3-*O*-sulfate	1.311	[M + H]^+^	381.9995	381.9968	−6.96	NA
28	Quercetin 3-(2″- glucosylgalactoside) 7-glucoside	79.839	[M + H]^+^	788.2011	788.2013	0.20	NA
*Isoflavonoids*							
29	Dalbergin	1.860	[M + H]^+^	268.0736	268.0725	−3.83	*Nostoc* sp. [5]
**Other polyphenols**							
*Phenolic glycoside*							
30	Dihydrocaffeic acid 3-*O*-glucuronide	1.394	[M + H]^+^	358.0900	358.0887	−3.60	*Nostoc* sp. [5]
31	5-(3′,5′-Dihydroxyphenyl)-gamma-valerolactone 3-*O*-glucuronide	1.611	[M + H]^+^	384.1056	384.1065	2.10	*Nostoc* sp. [5]
*Methoxyphenols*							
32	Curcumin I	1.444	[M + H]^+^	396.1573	396.1542	−7.88	NA
*Phloroglucinols*							
33	Phloroglucinol	1.494	[M + H]^+^	126.0317	126.0328	8.85	*Oscillatoria* sp., *Chroococcidiopsis* sp., *Leptolyngbya* sp., *Calothrix* sp., *Nostoc* sp. and *Phormidium* sp. [5,41]
34	Dihydrophloroglucinol	83.584	[M + H]^+^	128.0473	128.0469	−3.85	*Nostoc* sp. [5]
*Phenolic terpenes*							
35	Carnosic acid	82.418	[M + H]^+^	332.1988	332.1959	−8.68	*Nostoc* sp. [5]
36	11,12-Dimethylrosmanol	86.496	[M + H]^+^	374.2093	374.2088	−1.27	*Nostoc* sp. [5]
37	6,7-Dimethoxy-7-epirosmanol	86.496	[M + H]^+^	390.2042	390.2056	3.59	*Nostoc* sp. [5]
*Lignan derivatives*							
38	2′-Hydroxyenterolactone	84.599	[M + H]^+^	314.1154	314.1128	−8.29	NA

NA, not available.

**Table 3:** Comparison of identified compounds with previous studies on bioactivities.

No.	Proposed Compounds	Antioxidant Activity	Enzyme Inhibitory Activity	Anticancer Activity
**Phenolic acid**				
*Hydroxybenzoic acids*				
1	Vanillic acid 4-sulfate	NA	NA	NA
*Hydroxycinnamic acids*				
2	Cis-Caffeoyl tartaric acid	Pacifico et al. [42]; Cassino et al. [43]; Ghareeb et al. [44]	Li et al. [45]	Ghareeb et al. [44]
3	m-Coumaric acid	Sarker and Oba [46]; Roychoudhury et al. [47]	Roychoudhury et al. [47]	Roychoudhury et al. [47]
4	Cinnamic acid	Sova [48]; Pontiki et al. [49]	Adisakwattana et al. [50]; Sheng et al. [51]	Pontiki et al. [49]
5	2,5-Dimethoxycinnamic acid	NA	NA	NA
6	p-Methoxycinnamic acid ethyl ester	Rychlicka et al. [52]	Rychlicka et al. [52]; Płowuszyńska and Gliszczyńska [53]	Rychlicka et al. [52]
7	cis-Ferulic acid [arabinosyl-(1->3)-[glucosyl-(1->6)]-glucosyl] ester	NA	NA	NA
8	3,4,5-Trimethoxycinnamic acid	Zhao et al. [54]	Zhao et al. [54]; Kos et al. [55]	Zhao et al. [54]
*Hydroxyphenylpropanoic acids*				
9	Dihydrosinapic acid	Chen [56]; Nićiforović and Abramovič [57]	Chen [56]; Nićiforović and Abramovič [57]	Chen [56]; Nićiforović and Abramovič [57]
**Flavonoids**				
*Anthocyanins*				
10	Malvidin 3-sophoroside 5-glucoside	Ge et al. [58]	NA	Li et al. [59]; Mansour et al. [60]
11	Curcumin monoglucoside	Pandareesh et al. [61]	NA	Gurung et al. [62]
12	Malvidin 3-(6-coumaroylglucoside) 5-glucoside	NA	NA	NA
*Flavanols*				
13	(-)-Epicatechin 7-*O*-glucuronide	Natsume et al. [63]	NA	Widowati et al. [64]
14	7-Galloylcatechin	Plumb et al. [65]	Wu et al. [66]	Min and Kwon [67]
15	(±)-3′,4′-Methylenedioxy-5,7-dimethylepicatechin	Fan et al. [68]	NA	NA
16	Epicatechin 3-*O*-(4-methylgallate)	NA	NA	NA
17	3-methyl-epicatechin	Spencer et al. [69]	NA	NA
18	4′,7-Di-*O*-methylcatechin	NA	NA	NA
*Flavones*				
19	Luteolin 4′-glucoside 7-galacturonide	Park and Song [70]	Park and Song [70]	NA
20	6-Methoxyluteolin 7-glucuronide	Weng and Wang [71]	NA	NA
21	8-Hydroxyluteolin 4′-methyl ether 7-(6‴-acetylallosyl)(1->2)(6″-acetylglucoside)	NA	NA	NA
22	6-Hydroxyluteolin 6,3′-dimethyl ether 7,4′-disulfate	NA	NA	NA
23	6-Hydroxyluteolin 6,7- disulfate	NA	NA	NA
24	Luteolin 4′-methyl ether 7,3′-disulfate	NA	NA	NA
25	6-Hydroxyluteolin 4′-methyl eter 7-rhamnosyl-(1->2)-(6″-acetylglucoside)	NA	NA	NA
26	Luteolin 4′-methyl ether 7-(4G-rhamnosylneohesperidoside)	Cortés et al. [72]	Cortés et al. [72]	NA
*Flavonols*				
27	Quercetin 3-*O*-sulfate	Valentová et al. [73]; Gervasi et al. [74]	NA	Wu et al. [75]
28	Quercetin 3-(2″- glucosylgalactoside) 7-glucoside	NA	NA	NA
*Isoflavonoids*				
29	Dalbergin	Ma et al. [76]; Lucas et al. [77]	NA	Valojerdi et al. [78]; Khan et al. [79]
**Other polyphenols**				
*Phenolic glycoside*				
30	Dihydrocaffeic acid 3-*O*-glucuronide	Iqbal et al. [80]; Piazzon et al. [81]	NA	NA
31	5-(3′,5′-Dihydroxyphenyl)-gamma-valerolactone 3-*O*-glucuronide	Zhong et al. [82]	NA	NA
*Methoxyphenols*				
32	Curcumin I	Jakubczyk et al. [83]; Bisset et al. [84]	Bisset et al. [84]	Ojo et al. [85]
*Phloroglucinols*				
33	Phloroglucinol	Delfanian et al. [86]; Drygalski et al. [87]	Wan et al. [88]; Burmaoglu et al. [89]	Kim et al., [90]; Kang et al., [91]
34	Dihydrophloroglucinol	Zhang et al. [92]	NA	Chauthe et al. [93]
*Phenolic terpenes*				
35	Carnosic acid	Lin et al. [94]; Jiang et al. [95]	Sheng et al. [96]; Ercan and El [97]	Lin et al. [94]; Jiang et al. [95]
36	11,12-Dimethylrosmanol	Salem et al. [98]	Salem et al. [98]	Salem et al. [98]
37	6,7-Dimethoxy-7-epirosmanol	Salem et al. [98]	Salem et al. [98]	Salem et al. [98]
*Lignan derivatives*				
38	2′-Hydroxyenterolactone	Polat Kose and Gulcin [99]	NA	Mali et al. [100]

NA, not available.

## Data Availability

Not applicable.

## References

[1] Kumar Y., Tarafdar A., Kumar D., Saravanan C., Badgujar P.C., Pharande A., Pareek S., Fawole O.A. (2022). Polyphenols of edible macroalgae: Estimation of in vitro bio-accessibility and cytotoxicity, quantification by LC-MS/MS and potential utilization as an antimicrobial and functional food ingredient. Antioxidants.

[2] WHO (2003). Diet, Nutrition and the Prevention of Chronic Diseases.

[3] Barba-Ostria C., Carrera-Pacheco S.E., Gonzalez-Pastor R., Heredia-Moya J., Mayorga-Ramos A., Rodríguez-Pólit C., Zúñiga-Miranda J., Arias-Almeida B., Guamán L.P. (2022). Evaluation of biological activity of natural compounds: Current trends and methods. Molecules.

[4] Almendinger M., Saalfrank F., Rohn S., Kurth E., Springer M., Pleissner D. (2021). Characterization of selected microalgae and cyanobacteria as sources of compounds with antioxidant capacity. Algal Res..

[5] Lomakool S., Ruangrit K., Jeerapan I., Tragoolpua Y., Pumas C., Srinuanpan S., Pekkoh J., Duangjan K. (2021). Biological activities and phytochemicals profiling of different cyanobacterial and microalgal biomass. Biomass Conv. Biorefin..

[6] Pekkoh J., Lomakool S., Chankham J., Duangjan K., Thurakit T., Phinyo K., Ruangrit K., Tragoolpua Y., Pumas C., Pathom-aree W. (2022). Maximizing biomass productivity of cyanobacterium *Nostoc* sp. through high-throughput bioprocess optimization and application in multiproduct biorefinery towards a holistic zero waste. Biomass Conv. Biorefin..

[7] Dvořák P., Casamatta D.A., Hašler P., Jahodářová E., Norwich A.R., Poulíčková A., Hallenbeck P.C. (2017). Diversity of the cyanobacteria. Modern Topics in the Phototrophic Prokaryotes: Environmental and Applied Aspects.

[8] Guerreiro A., Andrade M.A., Menezes C., Vilarinho F., Dias E. (2020). Antioxidant and cytoprotective properties of cyanobacteria: Potential for biotechnological applications. Toxins.

[9] Martí-Quijal F.J., Ramon-Mascarell F., Pallarés N., Ferrer E., Berrada H., Phimolsiripol Y., Barba F.J. (2021). Extraction of antioxidant compounds and pigments from *Spirulina* (*Arthrospira Platensis*) assisted by pulsed electric fields and the binary mixture of organic solvents and water. Appl. Sci..

[10] Abdel-Raouf N., Ibraheem I.B., Abdel-Tawab S., Naser Y.A. (2011). Antimicrobial and antihyperlipidemic activities of isolated quercetin from *Anabaena aequalis*. J. Phycol..

[11] Costa M., Rosa F., Ribeiro T., Hernandez-Bautista R., Bonaldo M., Gonçalves Silva N., Eiríksson F., Thorsteinsdóttir M., Ussar S., Urbatzka R. (2019). Identification of cyanobacterial strains with potential for the treatment of obesity-related co-morbidities by bioactivity, toxicity evaluation and metabolite profiling. Mar. Drugs.

[12] Guldas M., Ziyanok-Demirtas S., Sahan Y., Yildiz E., Gurbuz O. (2020). Antioxidant and anti-diabetic properties of *Spirulina platensis* produced in Turkey. Food Sci. Technol..

[13] Yasin D., Zafaryab M., Ansari S., Ahmad N., Khan N.F., Zaki A., Rizvi M.M.A., Fatma T. (2019). Evaluation of antioxidant and anti-proliferative efficacy of *Nostoc muscorum* NCCU-442. Biocatal. Agric. Biotechnol..

[14] Pradhan B., Nayak R., Patra S., Bhuyan P.P., Dash S.R., Ki J.-S., Adhikary S.P., Ragusa A., Jena M. (2022). Cyanobacteria and algae-derived bioactive metabolites as antiviral agents: Evidence, mode of action, and scope for further expansion; a comprehensive review in light of the SARS-CoV-2 outbreak. Antioxidants.

[15] Tyagi S., Singh R.K., Tiwari S.P. (2021). Anti-enterococcal and anti-oxidative potential of a thermophilic cyanobacterium, *Leptolyngbya* sp. HNBGU 003. Saudi J. Biol. Sci..

[16] Tseng C.C., Yeh H.Y., Liao Z.H., Hung S.W., Chen B., Lee P.T., Nan F.H., Shih W.L., Chang C.C., Lee M.C. (2021). An in vitro study shows the potential of *Nostoc commune* (Cyanobacteria) polysaccharides extract for wound-healing and anti-allergic use in the cosmetics industry. J. Funct. Foods.

[17] Castaneda A., Ferraz R., Vieira M., Cardoso I., Vasconcelos V., Martins R. (2021). Bridging cyanobacteria to neurodegenerative diseases: A new potential source of bioactive compounds against Alzheimer’s disease. Mar. Drugs.

[18] Joshi D., Mohandass C., Mohandass C. (2017). Effect of UV-B radiation and desiccation stress on photoprotective compounds accumulation in marine *Leptolyngbya* sp.. Appl. Biochem. Biotechnol..

[19] Kokabi M., Yousefzadi M., Nejad Ebrahimi S., Soltani M., Malik S. (2022). Evaluating the photoprotective potential of *Leptolyngbya* sp.. Acta Physiol. Plant..

[20] Mahanil K., Sattayawat P., Pekkoh J., Kameya M., Ishii M., Pumas C. (2022). Simple transformation of the filamentous thermophilic cyanobacterium *Leptolyngbya* sp. KC45. Algal Res..

[21] Pumas C., Vacharapiyasophon P., Peerapornpisal Y., Leelapornpisid P., Boonchum W., Ishii M., Khanongnuch C. (2011). Thermostability of phycobiliproteins and antioxidant activity from four thermotolerant cyanobacteria. Phycol. Res..

[22] Rodriguez-Jasso R.M., Mussatto S.I., Pastrana L., Aguilar C.N., Teixeira J.A. (2011). Microwave-assisted extraction of sulfated polysaccharides (fucoidan) from brown seaweed. Carbohydr. Polym..

[23] Osório C., Machado S., Peixoto J., Bessada S., Pimentel F.B., Alves R.C., Oliveira M.B.P.P. (2020). Pigments content (chlorophylls, fucoxanthin and phycobiliproteins) of different commercial dried algae. Separations.

[24] Tang J., Dunshea F.R., Suleria H.A.R. (2020). LC-ESI-QTOF/MS characterization of phenolic compounds from medicinal plants (hops and juniper berries) and their antioxidant activity. Foods.

[25] Cheirsilp B., Wantip K., Chai-issarapap N., Maneechote W., Pekkoh J., Duangjan K., Ruangrit K., Pumas C., Pathom-aree W., Srinuanpan S. (2022). Enhanced production of astaxanthin and co-bioproducts from microalga *Haematococcus* sp. integrated with valorization of industrial wastewater under two-stage LED light illumination strategy. Environ. Technol. Innov..

[26] Ruangrit K., Chaipoot S., Phongphisutthinant R., Duangjan K., Phinyo K., Jeerapan I., Pekkoh J., Srinuanpan S. (2021). A successful biorefinery approach of macroalgal biomass as a promising sustainable source to produce bioactive nutraceutical and biodiesel. Biomass Conv. Biorefin..

[27] Pekkoh J., Ruangrit K., Pumas C., Duangjan K., Chaipoot S., Phongphisutthinant R., Jeerapan I., Sawangrat K., Pathom-aree W., Srinuanpan S. (2021). Transforming microalgal *Chlorella* biomass into cosmetically and nutraceutically protein hydrolysates using high-efficiency enzymatic hydrolysis approach. Biomass Conv. Biorefin..

[28] Thring T.S., Hili P., Naughton D.P. (2009). Anti-collagenase, anti-elastase and anti-oxidant activities of extracts from 21 plants. BMC Complement. Altern. Med..

[29] Pekkoh J., Phinyo K., Thurakit T., Lomakool S., Duangjan K., Ruangrit K., Pumas C., Jiranusornkul S., Yooin W., Cheirsilp B. (2022). Lipid profile, antioxidant and antihypertensive activity, and computational molecular docking of diatom fatty acids as ACE inhibitors. Antioxidants.

[30] Tanruean K., Poolprasert P., Suwannarach N., Kumla J., Lumyong S. (2021). Phytochemical analysis and evaluation of antioxidant and biological activities of extracts from three *Clauseneae* plants in Northern Thailand. Plants.

[31] Kaewkod T., Sangboonruang S., Khacha-Ananda S., Charoenrak S., Bovonsombut S., Tragoolpua Y. (2022). Combinations of traditional kombucha tea with medicinal plant extracts for enhancement of beneficial substances and activation of apoptosis signaling pathways in colorectal cancer cells. Food Sci. Technol..

[32] Akinmoladun A.C., Falaiye O.E., Ojo O.B., Adeoti A., Amoo Z.A., Olaleye M.T. (2022). Effect of extraction technique, solvent polarity, and plant matrix on the antioxidant properties of *Chrysophyllum albidum* G. Don (African Star Apple). Bull. Natl. Res. Cent..

[33] Dowlath M., Karuppannan S.K., Darul R., Mohamed K., Subramanian S., Arunachalam K.D. (2020). Effect of solvents on phytochemical composition and antioxidant activity of *Cardiospermum halicacabum* (L.) extracts. Pharmacogn. J..

[34] Xu Y., Harvey P.J. (2019). Carotenoid production by *Dunaliella salina* under red light. Antioxidants.

[35] Mohr R., Voβ B., Schliep M., Kurz T., Maldener I., Adams D.G., Larkum A.D.W., Chen M., Hess W.R. (2010). A new chlorophyll d-containing cyanobacterium: Evidence for niche adaptation in the genus *Acaryochloris*. ISME J..

[36] Jesumani V., Du H., Pei P., Aslam M., Huang N. (2020). Comparative study on skin protection activity of polyphenol-rich extract and polysaccharide-rich extract from *Sargassum vachellianum*. PLoS ONE.

[37] Aleixandre-Tudo J.L., Du Toit W., Solis-Oviedo R.L., De La Cruz Pech-Canul A. (2018). The role of UV-visible spectroscopy for phenolic compounds quantification in winemaking. Frontiers and New Trends in the Size of Fermented Food and Beverages.

[38] Gao E.B., Kyere-Yeboah K., Wu J., Qiu H. (2021). Photoautotrophic production of p-Coumaric acid using genetically engineered *Synechocystis* sp. Pasteur Culture Collection 6803. Algal Res..

[39] Kukil K., Lindberg P. (2022). Expression of phenylalanine ammonia lyases in *Synechocystis* sp. PCC 6803 and subsequent improvements of sustainable production of phenylpropanoids. Microb. Cell Fact..

[40] Pendyala B., Patras A. (2020). In silico Screening of Food Bioactive Compounds to Predict Potential Inhibitors of COVID-19 Main protease (Mpro) and RNA-dependent RNA polymerase (RdRp). ChemRxiv.

[41] Ijaz S., Hasnain S. (2016). Antioxidant potential of indigenous cyanobacterial strains in relation with their phenolic and flavonoid contents. Nat. Prod. Res..

[42] Pacifico S., D’Abrosca B., Scognamiglio M., Gallicchio M., Galasso S., Monaco P., Fiorentino A. (2013). Antioxidant polyphenolic constituents of *Vitis* × *labruscana* cv. ‘Isabella’. Open Nat. Prod. J..

[43] Cassino C., Gianotti V., Bonello F., Tsolakis C., Cravero M.C., Osella D. (2016). Antioxidant composition of a selection of italian red wines and their corresponding free-radical scavenging ability. J. Chem..

[44] Ghareeb M., Saad A., Ahmed W., Refahy L., Nasr S. (2018). HPLC-DAD-ESI-MS/MS characterization of bioactive secondary metabolites from *Strelitzia nicolai* leaf extracts and their antioxidant and anticancer activities in vitro. Pharmacogn. Res..

[45] Li W., Song Y., Sun W., Yang X., Liu X., Sun L. (2021). Both acidic pH value and binding interactions of tartaric acid with α-Glucosidase cause the enzyme inhibition: The Mechanism in α-Glucosidase inhibition of four caffeic and tartaric acid derivates. Front. Nutr..

[46] Sarker U., Oba S. (2020). Phenolic profiles and antioxidant activities in selected drought-tolerant leafy vegetable amaranth. Sci. Rep..

[47] Roychoudhury S., Sinha B., Choudhury B.P., Jha N.K., Palit P., Kundu S., Mandal S.C., Kolesarova A., Yousef M.I., Ruokolainen J. (2021). Scavenging properties of plant-derived natural biomolecule para-coumaric acid in the prevention of oxidative stress-induced diseases. Antioxidants.

[48] Sova M. (2012). Antioxidant and antimicrobial activities of cinnamic acid derivatives. Mini Rev. Med. Chem..

[49] Pontiki E., Hadjipavlou-Litina D., Litinas K., Geromichalos G. (2014). Novel cinnamic acid derivatives as antioxidant and anticancer agents: Design, synthesis and modeling studies. Molecules.

[50] Adisakwattana S., Moonsan P., Yibchok-Anun S. (2008). Insulin-releasing properties of a series of cinnamic acid derivatives in vitro and in vivo. J. Agric. Food Chem..

[51] Sheng Z., Ge S., Xu X., Zhang Y., Wu P., Zhang K., Xu X., Li C., Zhao D., Tang X. (2018). Design, synthesis and evaluation of cinnamic acid ester derivatives as mushroom tyrosinase inhibitors. MedChemComm.

[52] Rychlicka M., Rot A., Gliszczyńska A. (2021). Biological properties, health benefits and enzymatic modifications of dietary methoxylated derivatives of cinnamic acid. Foods.

[53] Płowuszyńska A., Gliszczyńska A. (2021). Recent developments in therapeutic and nutraceutical applications of p-methoxycinnamic acid from plant origin. Molecules.

[54] Zhao Z., Song H., Xie J., Liu T., Zhao X., Chen X., He X., Wu S., Zhang Y., Zheng X. (2019). Research Progress in the biological activities of 3,4,5-Trimethoxycinnamic acid (TMCA) derivatives. Eur. J. Med. Chem..

[55] Kos J., Strharsky T., Stepankova S., Svrckova K., Oravec M., Hosek J., Imramovsky A., Jampilek J. (2021). Trimethoxycinnamates and their cholinesterase inhibitory activity. Appl. Sci..

[56] Chen C. (2016). Sinapic acid and its derivatives as medicine in oxidative stress-induced diseases and aging. Oxid. Med. Cell. Longev..

[57] Nićiforović N., Abramovič H. (2014). Sinapic acid and its derivatives: Natural sources and bioactivity. Compr. Rev. Food Sci. Food Saf..

[58] Ge J., Hu Y., Wang H., Huang Y., Zhang P., Liao Z., Chen M. (2017). Profiling of anthocyanins in transgenic purple-fleshed sweet potatoes by HPLC-MS/MS: Profiling of anthocyanins in transgenic purple-fleshed sweet potatoes. J. Sci. Food Agric..

[59] Li D., Wang P., Luo Y., Zhao M., Chen F. (2017). Health benefits of anthocyanins and molecular mechanisms: Update from recent decade. Crit. Rev. Food Sci. Nutr..

[60] Mansour K.A., Moustafa S.F., Abdelkhalik S.M. (2021). High-Resolution UPLC-MS profiling of anthocyanins and flavonols of red cabbage (*Brassica oleracea* L. var. capitata f. rubra DC.) cultivated in Egypt and evaluation of their biological activity. Molecules.

[61] Pandareesh M.D., Shrivash M.K., Naveen K.H.N., Misra K., Srinivas B.M.M. (2016). curcumin monoglucoside shows improved bioavailability and mitigates rotenone induced neurotoxicity in cell and drosophila models of Parkinson’s disease. Neurochem. Res..

[62] Gurung R.B., Gong S.Y., Dhakal D., Le T.T., Jung N.R., Jung H.J., Oh T.J., Sohng J.K. (2017). Synthesis of curcumin glycosides with enhanced anticancer properties using one-pot multienzyme glycosylation technique. J. Microbiol. Biotechnol..

[63] Natsume M., Osakabe N., Yasuda A., Baba S., Tokunaga T., Kondo K., Osawa T., Terao J. (2004). In vitro antioxidative activity of (−)-epicatechin glucuronide metabolites present in human and rat plasma. Free Radic. Res..

[64] Widowati W., Jasaputra D.K., Heriady Y., Faried A., Rizal R., Widodo W.S., Benowo Wibowo S.H., Kusuma H.S.W., Girsang E., Ehrich Lister I.N. (2019). Dietary flavonoids against various breast cancer subtypes: A molecular docking study. ScienceAsia.

[65] Plumb G.W., de Pascual-Teresa S., Santos-Buelga C., Rivas-Gonzalo J.C., Williamson G. (2002). Antioxidant properties of gallocatechin and prodelphinidins from pomegranate peel. Redox Rep..

[66] Wu X., Ding H., Hu X., Pan J., Liao Y., Gong D., Zhang G. (2018). Exploring inhibitory mechanism of gallocatechin gallate on a-amylase and a-glucosidase relevant to postprandial hyperglycemia. J. Funct. Foods.

[67] Min K.J., Kwon T.K. (2014). Anticancer effects and molecular mechanisms of epigallocatechin-3-gallate. Integr. Med. Res..

[68] Fan M., Lee J.I., Ryu Y.B., Choi Y.J., Tang Y., Oh M., Kim E.K. (2021). Comparative analysis of metabolite profiling of *Momordica charantia* leaf and the anti-obesity effect through regulating lipid metabolism. Int. J. Env. Res. Pub. Health.

[69] Spencer J.P.E., Schroeter H., Rechner A.R., Rice-Evans C. (2001). Bioavailability of flavan-3-ols and procyanidins: Gastrointestinal tract influences and their relevance to bioactive forms in vivo. Antioxid. Redox Signal..

[70] Park C.M., Song Y.-S. (2019). Luteolin and luteolin-7-O-glucoside protect against acute liver injury through regulation of inflammatory mediators and antioxidative enzymes in GalN/LPS-induced hepatitic ICR mice. Nutr. Res. Pract..

[71] Weng X.C., Wang W. (2000). Antioxidant activity of compounds isolated from *Salvia plebeia*. Food Chem..

[72] Cortés C., González-Cabrera D.A., Barrientos R., Parra C., Romero-Parra J., Pertino M.W., Areche C., Sepúlveda B., Bórquez J., Torres-Benítez A. (2022). Phenolic profile, antioxidant and enzyme inhibition properties of the chilean endemic plant *Ovidia pillopillo* (Gay) meissner (Thymelaeaceae). Metabolites.

[73] Valentová K., Káňová K., Di Meo F., Pelantová H., Chambers C.S., Rydlová L., Petrásková L., Křenková A., Cvačka J., Trouillas P. (2017). Chemoenzymatic preparation and biophysical properties of sulfated quercetin. Metab. Int. J. Mol. Sci..

[74] Gervasi T., Calderaro A., Barreca D., Tellone E., Trombetta D., Ficarra S., Smeriglio A., Mandalari G., Gattuso G. (2022). Biotechnological applications and health-promoting properties of flavonols: An updated view. Int. J. Mol. Sci..

[75] Wu Q., Needs P.W., Lu Y., Kroon P.A., Ren D., Yang X. (2018). Different antitumor effects of quercetin, quercetin-3′-sulfate and quercetin-3-glucuronide in human breast cancer MCF-7 cells. Food Funct..

[76] Ma R.K., Luo J., Qiao M.J., Fu Y.L., Zhu P.C., Wei P.L., Liu Z.G. (2022). Chemical composition of extracts from *Dalbergia odorifera* heartwood and its correlation with color. Ind. Crop. Prod..

[77] Lucas C.I.S., Ferreira A.F., Costa M.A.P.C., Silva F.L., Estevinho L.M., Carvalho C.A.L. (2020). Phytochemical study and antioxidant activity of *Dalbergia ecastaphyllum*. Rodriguésia.

[78] Mahdizade V.F., Goliaei B., Parivar K., Nikoofar A. (2019). Effect of a neoflavonoid (Dalbergin) on T47D breast cancer cell line and mRNA levels of *p53*, *Bcl*-2, and *STAT3* Genes. Iran. Red Crescent Med. J..

[79] Khan A.U., Dagur H.S., Khan M., Malik N., Alam M., Mushtaque M. (2021). Therapeutic role of flavonoids and flavones in cancer prevention: Current trends and future perspectives. Eur. J. Med. Chem. Rep..

[80] Iqbal Y., Ponnampalam E.N., Suleria H.A.R., Cottrell J.J., Dunshea F.R. (2021). LC-ESI/QTOF-MS profiling of Chicory and Lucrene polyphenols and their antioxidant activities. Antioxidants.

[81] Piazzon A., Vrhovsek U., Masuero D., Mattivi F., Mandoj F., Nardini M. (2012). Antioxidant activity of phenolic acids and their metabolites: Synthesis and antioxidant properties of the sulfate derivatives of ferulic and caffeic acids and of the acyl glucuronide of ferulic acid. J. Agric. Food Chem..

[82] Zhong B., Robinson N.A., Warner R.D., Barrow C.J., Dunshea F.R., Suleria H.A.R. (2020). LC-ESI-QTOF-MS/MS characterization of seaweed phenolics and their antioxidant potential. Mar. Drugs.

[83] Jakubczyk K., Drużga A., Katarzyna J., Skonieczna-żydecka K. (2020). Antioxidant potential of curcumin—A meta-analysis of randomized clinical trials. Antioxidants.

[84] Bisset S., Sobhi W., Bensouici C., Khenchouche A. (2022). Antioxidant Activity and Inhibitory Effect of Curcumin on Some Enzymes Involved in Several Diseases: Acetylcholinesterase, Butyrylcholinesterase, α-Glucosidase and Tyrosinase.

[85] Ojo O.A., Adeyemo T.R., Rotimi D., Batiha G.E.-S., Mostafa-Hedeab G., Iyobhebhe M.E., Elebiyo T.C., Atunwa B., Ojo A.B., Lima C.M.G. (2022). Anticancer properties of curcumin against colorectal cancer: A review. Front. Oncol..

[86] Delfanian M., Sahari M.A., Barzegar M., Ahmadi Gavlighi H. (2021). Structure–antioxidant activity relationships of gallic acid and phloroglucinol. J. Food Meas. Charact..

[87] Drygalski K., Siewko K., Chomentowski A., Odrzygóźdź C., Zalewska A., Krętowski A., Maciejczyk M. (2021). Phloroglucinol strengthens the antioxidant barrier and reduces oxidative/nitrosative stress in nonalcoholic fatty liver disease (NAFLD). Oxid. Med. Cell. Longev..

[88] Wan J.-X., Lim G., Lee J., Sun X.-B., Gao D.-Y., Si Y.-X., Shi X.-L., Qian G.-Y., Wang Q., Park Y.-D. (2019). Inhibitory effect of phloroglucinol on α-glucosidase: Kinetics and molecular dynamics simulation integration study. Int. J. Biol. Macromol..

[89] Burmaoglu S., Yilmaz A.O., Taslimi P., Algul O., Kılıç D., Gulcin I. (2018). Synthesis and biological evaluation of phloroglucinol derivatives possessing α-glycosidase, acetylcholinesterase, butyrylcholinesterase, carbonic anhydrase inhibitory activity. Arch. Pharm..

[90] Kim R.-K., Uddin N., Hyun J.-W., Kim C., Suh Y., Lee S.-J. (2015). Novel anticancer activity of phloroglucinol against breast cancer stem-like cells. Toxicol. Appl. Pharmacol..

[91] Kang M.H., Kim I.H., Nam T.J. (2014). Phloroglucinol induces apoptosis via apoptotic signaling pathways in HT-29 colon cancer cells. Oncol. Rep..

[92] Zhang S., Hu C.Y., Guo Y., Wang X., Meng Y. (2021). Polyphenols in fermented apple juice: Beneficial effects on human health. J. Funct. Foods.

[93] Chauthe S.K., Bharate S.B., Periyasamy G., Khanna A., Bhutani K.K., Mishra P.D., Singh I.P. (2012). One pot synthesis and anticancer activity of dimeric phloroglucinols. Bioorg. Med. Chem. Lett..

[94] Lin K.I., Lin C.C., Kuo S.M., Lai J.C., Wang Y.Q., You H.L., Hsu M.L., Chen C.H., Shiu L.Y. (2018). Carnosic acid impedes cell growth and enhances anticancer effects of carmustine and lomustine in melanoma. Biosci. Rep..

[95] Jiang S., Qiu Y., Wang Z., Ji Y., Zhang X., Yan X., Zhan Z. (2021). Carnosic acid induces antiproliferation and anti-metastatic property of esophageal cancer cells via MAPK signaling pathways. J. Oncol..

[96] Sheng Z., Ai B., Zheng L., Zheng X., Xu Z., Shen Y., Jin Z. (2018). Inhibitory activities of kaempferol, galangin, carnosic acid and polydatin against glycation and α-amylase and α-glucosidase enzymes. Food Sci. Technol..

[97] Ercan P., El S.N. (2018). Bioaccessibility and inhibitory effects on digestive enzymes of carnosic acid in sage and rosemary. Int. J. Biol. Macromol..

[98] Salem M.A., Radwan R.A., Mostafa E.S., Alseekh S., Fernie A.R., Ezzat S.M. (2020). Using an UPLC/MS-based untargeted metabolomics approach for assessing the antioxidant capacity and anti-aging potential of selected herbs. RSC Adv..

[99] Polat Kose L., Gulcin I. (2021). Evaluation of the antioxidant and antiradical properties of some phyto and mammalian lignans. Molecules.

[100] Mali A.V., Padhye S.B., Anant S., Hegde M.V., Kadam S.S. (2019). Anticancer and antimetastatic potential of enterolactone: Clinical, preclinical and mechanistic perspectives. Eur. J. Pharmacol..

[101] Darvin M.E., Lademann J., von Hagen J., Lohan S.B., Kolmar H., Meinke M.C., Jung S. (2022). Carotenoids in human skin in vivo: Antioxidant and photo-protectant role against external and internal stressors. Antioxidants.

[102] Anas A., Vinothkumar S., Gupta S., Jasmin C., Joseph V., Parameswaran P.S., Nair S. (2016). Evaluation of antioxidant and cytotoxic properties of *Cynobacteria, Limnothrix* sp. and *Leptolyngbya* sp. from Arabian sea. J. Microbiol. Biotech. Food Sci..

[103] Mali D., Ratnaparkhe S., Nabar B. (2022). In Vitro Study of the Anti-inflammatory and Antioxidant Activity of the *Leptolyngbya* spp. Isolated from Lonar Lake. Int. J. Appl. Pharm. Sci. Res..

[104] Singh D.P., Prabha R., Verma S., Meena K.K., Yandigeri M. (2017). Antioxidant properties and polyphenolic content in terrestrial cyanobacteria. 3 Biotech.

[105] Raharjo S., Fatanah V.N., Susilaningsih D., Kasim M., Susilawati P.E., Rahman D.Y. (2019). Screening of marine microalgae collected from Wakatobi as anti-tyrosinase. J. Phys.Conf. Ser..

[106] Sahin S.C. (2018). The potential of *Arthrospira platensis* extract as a tyrosinase inhibitor for pharmaceutical or cosmetic applications. S. Afr. J. Bot..

[107] Chaiyana W., Sirithunyalug J., Somwongin S., Punyoyai C., Laothaweerungsawat N., Marsup P., Neimkhum W., Yawootti A. (2020). Enhancement of the antioxidant, anti-tyrosinase, and anti-hyaluronidase activity of *Morus alba* L. leaf extract by pulsed electric field extraction. Molecules.

[108] Klomsakul P., Chalopagorn P. (2019). In vitro antioxidant activity, inhibitory effect of tyrosinase and DOPA auto-oxidation by *Wrightia religiosa* extracts. S. Afr. J. Bot..

[109] Zuo A.R., Cao S.W., Zuo A.R., Dong H.H., Shu Q.L., Zheng L.X., Yu X.Y., Yu Y.Y., Cao S.W. (2018). The antityrosinase and antioxidant activities of flavonoids dominated by the number and location of phenolic hydroxyl groups. Chin. Med..

[110] Karim A.A., Azlan A., Ismail A., Hashim P., Abd Gani S.S., Zainudin B.H., Abdullah N.A. (2014). Phenolic composition, antioxidant, anti-wrinkles and tyrosinase inhibitory activities of cocoa pod extract. BMC Complement. Altern. Med..

[111] Jiamphun S., Chaiyana W. (2022). Enhanced antioxidant, hyaluronidase, and collagenase inhibitory activities of glutinous rice husk extract by aqueous enzymatic extraction. Molecules.

[112] Nitthikan N., Leelapornpisid P., Naksuriya O., Intasai N., Kiattisin K. (2022). Potential and alternative bioactive compounds from brown *Agaricus bisporus* mushroom extracts for xerosis treatment. Sci. Pharm..

[113] Jiratchayamaethasakul C., Ding Y., Hwang O., Im S.-T., Jang Y., Myung S.-W., Lee J.M., Kim H.-S., Ko S.-C., Lee S.-H. (2020). In vitro screening of elastase, collagenase, hyaluronidase, and tyrosinase inhibitory and antioxidant activities of 22 halophyte plant extracts for novel cosmeceuticals. Fish. Aquat. Sci..

[114] Nurrochmad A., Wirasti W., Dirman A., Lukitaningsih E., Rahmawati A., Fakhrudin N. (2018). Effects of antioxidant, anti-collagenase, anti-elastase, anti-tyrosinase of the extract and fraction from *Turbinaria decurrens* Bory. Indones. J. Pharm..

[115] Eun C.-H., Kang M.-S., Kim I.-J. (2020). Elastase/collagenase inhibition compositions of *Citrus unshiu* and its association with phenolic content and anti-oxidant activity. Appl. Sci..

[116] Mechqoq H., Hourfane S., El Yaagoubi M., El Hamdaoui A., da Silva Almeida J.R.G., Rocha J.M., El Aouad N. (2022). Molecular docking, tyrosinase, collagenase, and elastase inhibition activities of argan by-products. Cosmetics.

[117] Ali M.Y., Seong S.H., Jung H.A., Choi J.S. (2019). Angiotensin-I-converting enzyme inhibitory activity of coumarins from *Angelica decursiva*. Molecules.

[118] Mirzaei M., Mirdamadi S., Safavi M. (2020). Structural analysis of ACE-inhibitory peptide (VL-9) derived from *Kluyveromyces marxianus* protein hydrolysate. J. Mol. Struct..

[119] Kheeree N., Sangtanoo P., Srimongkol P., Saisavoey T., Reamtong O., Choowongkomon K., Karnchanatat A. (2020). ACE inhibitory peptides derived from de-fatted lemon basil seeds: Optimization, purification, identification, structure-activity relationship and molecular docking analysis. Food Funct..

[120] Liu B., Yang J., Ma Y., Yuan E., Chen C. (2010). Antioxidant and angiotensin converting enzyme (ACE) inhibitory activities of ethanol extract and pure flavonoids from *Adinandra nitida* leaves. Pharm. Biol..

[121] Sukandar E.Y., Sutjiatmo A.B., Vikasari S.N. Angiotensin converting enzyme inhibitor activity of ethanol extract of *Sonchus arvensis* (Linn.) leaves. Proceedings of the 6th International Conference on Bioinformatics and Biomedical Science 2017.

[122] Al Shukor N., Van Camp J., Gonzales G.B., Staljanssens D., Struijs K., Zotti M.J., Raes K., Smagghe G. (2013). Angiotensin-converting enzyme inhibitory effects by plant phenolic compounds: A study of structure activity relationships. J. Agric. Food Chem..

[123] Tan Y., Chang S.K.C., Zhang Y. (2017). Comparison of α-amylase, α-glucosidase and lipase inhibitory activity of the phenolic substances in two black legumes of different genera. Food Chem..

[124] Buzgaia N., Lee S.Y., Rukayadi Y., Abas F., Shaari K. (2021). Antioxidant activity, α-glucosidase inhibition and UHPLC–ESI–MS/MS profile of shmar (*Arbutus pavarii* Pamp). Plants.

[125] Pinaffi A.C., Sampaio G.R., Soares M.J., Shahidi F., de Camargo A.C., Torres E.A.F.S. (2020). Insoluble-bound polyphenols released from guarana powder: Inhibition of alpha-glucosidase and proanthocyanidin profile. Molecules.

[126] Gradíssimo D.G., Oliveira da Silva V.C., Xavier L.P., do Nascimento S.V., Valadares R.B.D.S., Faustino S.M.M., Schneider M.P.C., Santos A.V. (2021). Glucosidase inhibitors screening in microalgae and cyanobacteria isolated from the amazon and proteomic analysis of inhibitor producing *Synechococcus* sp. GFB01. Microorganisms.

[127] Jadalla B.M., Moser J.J., Sharma R., Etsassala N.G., Egieyeh S.A., Badmus J.A., Marnewick J.L., Beukes D., Cupido C.N., Hussein A.A. (2022). In vitro alpha-glucosidase and alpha-amylase inhibitory activities and antioxidant capacity of *Helichrysum cymosum* and *Helichrysum pandurifolium* Schrank constituents. Separations.

[128] Proença C., Freitas M., Ribeiro D., Oliveira E.F.T., Sousa J.L.C., Tomé S.M., Ramos M.J., Silva A.M.S., Fernandes P.A., Fernandes E. (2017). α-Glucosidase inhibition by flavonoids: An in vitro and in silico structure–activity relationship study. J. Enzym. Inhib. Med. Chem..

[129] Ahn J.H., Ryu S.H., Lee S., Yeon S.W., Turk A., Han Y.K., Lee K.Y., Hwang B.Y., Lee M.K. (2021). Aromatic constituents from the leaves of *Actinidia arguta* with antioxidant and α-glucosidase inhibitory activity. Antioxidants.

[130] Etsassala N., Badmus J.A., Marnewick J.L., Egieyeh S., Iwuoha E.I., Nchu F., Hussein A.A. (2022). Alpha-glucosidase and alpha-amylase inhibitory activities, molecular docking, and antioxidant capacities of *Plectranthus ecklonii* constituents. Antioxidants.

[131] Trabelsi L., Chaieb O., Mnari A., Abid-Essafi S., Aleya L. (2016). Partial characterization and antioxidant and antiproliferative activities of the aqueous extracellular polysaccharides from the thermophilic microalgae *Graesiella* sp.. BMC Complement. Altern. Med..

[132] Peña-Morán O.A., Villarreal M.L., Álvarez-Berber L., Meneses-Acosta A., Rodríguez-López V. (2016). Cytotoxicity, Post-treatment recovery, and selectivity analysis of naturally occurring podophyllotoxins from *Bursera fagaroides* var. fagaroides on breast cancer cell lines. Molecules.

[133] Calderón-Montaño J.M., Martínez-Sánchez S.M., Jiménez-González V., Burgos-Morón E., Guillén-Mancina E., Jiménez-Alonso J.J., Díaz-Ortega P., García F., Aparicio A., López-Lázaro M. (2021). Screening for selective anticancer activity of 65 extracts of plants collected in western Andalusia, Spain. Plants.

[134] Weerapreeyakul N., Nonpunya A., Barusrux S., Thitimetharoch T., Sripanidkulchai B. (2012). Evaluation of the anticancer potential of six herbs against a Hepatoma cell line. Chin. Med..

[135] Rashidi M., Seghatoleslam A., Namavari M., Amiri A., Fahmidehkar M.A., Ramezani A., Eftekhar E., Hosseini A., Erfani N., Fakher S. (2017). Selective cytotoxicity and apoptosis-induction of *Cyrtopodion scabrum* extract against digestive cancer cell lines. Int. J. Cancer Manag..

[136] Krzywik J., Mozga W., Aminpour M., Janczak J., Maj E., Wietrzyk J., Tuszyński J.A., Huczyński A. (2020). Synthesis, antiproliferative activity and molecular docking studies of novel doubly modified colchicine amides and sulfonamides as anticancer agents. Molecules.

[137] Nurgali K., Jagoe R.T., Abalo R. (2018). Editorial: Adverse effects of cancer chemotherapy: Anything new to improve tolerance and reduce sequelae?. Front. Pharmacol..

[138] Karan T., Aydin A. (2018). Anticancer potential and cytotoxic effect of some freshwater cyanobacteria. Trop. J. Pharm. Res..

[139] Kim A., Im M., Gu M.J., Ma J.Y. (2016). Citrus unshiu peel extract alleviates cancer-induced weight loss in mice bearing CT-26 adenocarcinoma. Sci. Rep..

[140] Mesas C., Martínez R., Ortiz R., Quinonero F., Prados J., Porres J.M., Melguizo C. (2021). Antioxidant and antiproliferative potential of ethanolic extracts from *Moringa oleifera*, *Tropaeolum tuberosum* and *Annona cherimola* in colorrectal cancer cells. Biomed. Pharmacother..

[141] N’guessan B.B., Asiamah A.D., Arthur N.K., Frimpong-Manso S., Amoateng P., Amponsah S.K., Kukuia K.E., Sarkodie J.A., Opuni K.F.M., Asiedu-Gyekye I.J. (2021). Ethanolic extract of *Nymphaea lotus* L. (Nymphaeaceae) leaves exhibits in vitro antioxidant, in vivo anti-inflammatory and cytotoxic activities on Jurkat and MCF-7 cancer cell lines. BMC Complement. Altern. Med..

[142] Sangtitanu T., Sangtanoo P., Srimongkol P., Saisavoey T., Reamtong O., Karnchanatat A. (2020). Peptides obtained from edible mushrooms: *Hericium erinaceus* offers the ability to scavenge free radicals and induce apoptosis in lung cancer cells in humans. Food Funct..

[143] Suantai B., Jantakee K., Kaewkod T., Sangboonruang S., Chitov T., Tragoolpua Y. (2022). Anthocyanins in red jasmine rice (*Oryza sativa* L.) extracts and efficacy on inhibition of herpes simplex virus, free radicals and cancer cell. Nutrients.

[144] Fu Y.B., Ahmed Z., Yang H., Horbach C. (2018). TUNEL Assay and DAPI staining revealed few alterations of cellular morphology in naturally and artificially aged seeds of cultivated flax. Plants.

